# Engineering PdAu/CeO_2_ Alloy/Oxide Interfaces for Selective Methane‐to‐Methanol Conversion with Water

**DOI:** 10.1002/anie.202505716

**Published:** 2025-07-24

**Authors:** Estefanía Fernández‐Villanueva, Pedro J. Ramírez, Pablo G. Lustemberg, Rubén Pérez, M. Verónica Ganduglia‐Pirovano, José A. Rodriguez

**Affiliations:** ^1^ Universitat Politècnica de València Camí de Vera s/n Valencia 46022 Spain; ^2^ Instituto de Catálisis y Petroleoquímica (ICP‐CSIC) C/ de Marie Curie 2 Madrid 28049 Spain; ^3^ Departamento de Física Teórica de la Materia Condensada Universidad Autónoma de Madrid Ciudad Universitaria de Cantoblanco Madrid E‐28049 Spain; ^4^ Facultad de Ciencias Universidad Central de Venezuela Caracas Distrito Capital 1020‐A Venezuela; ^5^ Zoneca‐CENEX R&D Laboratories Alta Vista Monterrey 64770 Mexico; ^6^ Condensed Matter Physics Center (IFIMAC) Universidad Autónoma de Madrid Ciudad Universitaria de Cantoblanco Madrid E‐28049 Spain; ^7^ Chemistry Division Brookhaven National Laboratory Upton New York 11973 USA

**Keywords:** Alloy–oxide interface, DFT calculations, Methane‐to‐methanol conversion, PdAu/CeO_2_ catalyst, XPS characterization

## Abstract

The direct conversion of methane‐to‐methanol remains a critical challenge in methane valorization. In this study, we unveil the crucial role of PdAu/CeO_2_ catalysts in enabling selective methane transformation under mild conditions, using only water as the sole oxidant. Through a combination of experimental techniques, including XPS and catalytic testing, alongside density functional theory (DFT) calculations, we demonstrate that a Pd_0.3_Au_0.7_/CeO_2_ catalyst, which predominantly exposes isolated Pd atoms, achieves remarkable methanol selectivity (∼80%) at 500 K with a 1:1 methane‐to‐water ratio. While Pd/CeO_2_ efficiently activates methane, its tendency for overreaction leads to complete methanol decomposition, thereby limiting selectivity. Alloying Pd with Au on ceria mitigates this over‐reactivity, preventing methanol degradation while maintaining sufficient catalytic activity. The PdAu/CeO_2_ composite exhibits a synergistic effect: Pd in contact with the ceria support facilitates methane activation and water dissociation, while Au fine‐tunes reactivity to promote methanol formation. DFT calculations confirm that isolated Pd sites at the PdAu/CeO_2_ interface play a key role in balancing activity and selectivity. This work underscores the importance of alloy/oxide interfaces in controlling selective methane conversion with water and offers valuable insights for designing highly efficient catalysts for methanol synthesis.

## Graphical abstract

Selective methane‐to‐methanol conversion at 500 K over Pd_0.3_Au_0.7_/CeO_2_. The synergistic interaction between alloyed Pd–Au sites and the CeO_2_ support enhances catalytic performance, enabling highly selective methane‐to‐methanol under mild conditions. The reaction of CH_4_ with H_2_O produces CH_3_OH and H_2_, achieving ∼80% selectivity under mild conditions while maintaining stable long‐term activity.

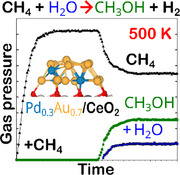

## Introduction

The selective conversion of methane‐to‐methanol is a crucial step in the valorization of natural gas.^[^ ^1^, ^2^, ^3^ ^]^ Numerous catalytic and noncatalytic approaches have been explored to achieve a direct CH_4_ → CH_3_OH transformation, aiming to bypass the energy‐intensive two‐step syngas route (CH_4_ → CO + H_2_ → CH_3_OH).^[^ ^4^, ^5^, ^6^, ^7^, ^8^, ^9^, ^10^, ^11^, ^12^, ^13^, ^14^ ^]^ One promising strategy involves the selective oxidation of methane using hydrogen peroxide (H_2_O_2_) as a key reactant,^[^ ^9^, ^15^ ^]^ where Pd and Pd–Au alloy catalysts have demonstrated significant activity.^[^ ^9^, ^10^, ^16^, ^17^, ^18^ ^]^ However, the direct oxidation of methane with water or hydroxyl (OH) groups remains highly attractive yet extremely challenging.^[^ ^12^, ^13^, ^15^, ^16^, ^19^, ^20^, ^21^ ^]^ In this study, we investigate several fundamental phenomena governing the conversion of methane‐to‐methanol using water as the oxidant. The three main competing reactions, along with their thermodynamics, are as follows:^[^ ^22^ ^]^

(1) $${\mathrm{CH}}_{4}+H_{2}O\to \;{\mathrm{CH}}_{3}\mathrm{OH}+H_{2},\;\Delta H_{\mathrm{reaction}}=113\;\mathrm{kj}\mathrm{mol}$$

(2) $$CH_{4}+H_{2}O\to \mathrm{CO}+3H_{2},\;\Delta H_{\mathrm{reaction}}=208\;\mathrm{kj}\mathrm{mol}$$

(3) $$CH_{4}+2H_{2}O\to CO_{2}+4H_{2},\;\Delta H_{\mathrm{reaction}}=165\;\mathrm{kj}\mathrm{mol}$$

The desired pathway is reaction (1), which, although endothermic, is feasible at 500−550 K. However, it competes with reactions (2) and (3), which are both more endothermic. Notably, reactions (1) and (2) proceed with a 1:1 methane‐to‐water ratio, whereas reaction (3) occurs at a 1:2 ratio. Consequently, maintaining a 1:1 CH_4_:H_2_O ratio may favor methanol formation at medium temperatures.

Another key factor influencing selectivity is the relative stability of the C─H bond in methane versus the C─O bond in the CH_3_O intermediate, with the following thermodynamic values:^[^ ^21^ ^]^

(4) $$CH_{4}\to CH_{3}+H,\;\Delta H_{\mathrm{reaction}}=439\;\mathrm{kj}\;\mathrm{mol}$$

(5) $$CH_{3}\to CH_{3}+O,\;\Delta H_{\mathrm{reaction}}=367\;\mathrm{kj}\mathrm{mol}$$

The Pd/CeO_2_ system is known to activate methane even at room temperature,^[^ ^9^ ^]^ but Pd tends to over‐oxidize methoxy and methanol intermediates, leading to their decomposition.^[^ ^23^ ^]^ A potential solution to mitigate this over‐reactivity is alloying Pd with an inert metal such as gold.^[^ ^24^, ^25^, ^26^ ^]^

In this work, we prepared a series of Pd*_x_* Au_1‐_ *_x_* and Pd*_x_* Au_1‐_ *_x_*/CeO_2_ systems (*x* = 0.3–0.5) and systematically investigated their reactivity toward CH_4_, H_2_O, and CH_3_OH using X‐ray photoelectron spectroscopy (XPS), catalytic testing, and density functional theory (DFT) calculations. While Pd*_x_* Au_1‐_ *_x_* alloys prevent methanol decomposition, these bimetallic systems alone do not activate methane or water efficiently at low temperatures. In contrast, Pd/CeO_2_(111) facilitates methane activation but also promotes methanol decomposition, making it highly effective for reactions (2) and (3) but suboptimal for reaction (1). To achieve an optimal balance between activity and selectivity for methanol formation, the presence of a PdAu‐CeO_2_ interface is essential, along with a precise control of the methane‐to‐water ratio in the reaction feed. Our engineered Pd_0.3_Au_0.7_/CeO_2_(111) composite effectively activates methane while preserving CH_3_O, making it a promising catalyst for reaction (1). At 500 K, it achieves methanol selectivity of nearly 80% with a 1:1 methane‐to‐water ratio and 30% with a 1:2 ratio. In the PdAu/CeO_2_ composite, each component has a distinct role, generating a synergistic effect that is absent in the individual binary catalysts (PdAu, Pd/CeO_2_, or Au/CeO_2_). This synergy is critical for enabling an efficient CH_4_ → CH_3_OH transformation with water as the sole oxidant.

## Results and Discussion

### Preparation of PdAu and PdAu/CeO_2_(111) Systems

In the following sections, we examine how the chemical and catalytic properties of palladium can be fine‐tuned through Pd–Au alloy formation. These alloy systems were prepared following methodologies previously reported in the literature,^[^ ^27^, ^28^ ^]^ with details on synthesis and characterization provided in the Supporting Information (Figures S1–S6). The primary challenge was to identify alloy compositions that prevent methanol decomposition. To address this, we investigated the adsorption and decomposition behavior of CH_3_OH on PdAu films with varying compositions, created by vapor deposition of Pd and Au onto a Mo(110) substrate.^[^ ^27^ ^]^ As discussed below, the optimal balance between effective methanol adsorption and minimal decomposition was achieved with a Pd_0.3_Au_0.7_ alloy. Core‐level spectra of this alloy exhibited binding energies and a line shape characteristic of Pd–Au bimetallic systems (Figure S1).^[^ ^27^, ^29^ ^]^ Low‐energy ion scattering spectroscopy (LEISS), using incident He^+^ ions, revealed that the alloy surface contained only 19 ± 2% Pd. This result is consistent with the well‐known tendency of Au to segregate to the surface of PdAu alloys,^[^ ^27^, ^30^ ^]^ a phenomenon attributed to the lower surface free energy of Au compared to Pd.^[^ ^31^ ^]^

Results of scanning tunneling microscopy (STM) indicate that Pd and Au grow on fully oxidized CeO_2_(111) forming 3D nanoparticles.^[^ ^32^, ^33^ ^]^ The interaction of Au with CeO_2_(111) is weak and this admetal preferentially grows on defects of the surface^[^ ^33^ ^]^ or on sites where another metal sits.^[^ ^34^ ^]^ To generate PdAu alloys on a CeO_2_(111) support, we employed sequential deposition of Pd and Au, with Pd deposited first to create nucleation sites for Au on the oxide surface.^[^ ^34^ ^]^ The final PdAu/CeO_2_(111) system was annealed to 600 K, and alloy formation was monitored via XPS (Figures S2 and S3), LEISS (Figure S4), and CO adsorption as a probe molecule (Figures S5 and S6). Following the deposition of 0.12 ML of Pd, the Pd 3d XPS spectrum indicated the presence of Pd^δ+^ centers on the ceria surface.^[^ ^35^ ^]^ Subsequent deposition of 0.28 ML of Au, followed by annealing to 600 K, led to spectral changes in the Pd 3d (Figure S2) and Au 4f (Figure S3) regions, confirming PdAu alloy formation.^[^ ^27^, ^29^ ^]^ Bimetallic bonding induces electronic perturbations that lead to complex shifts in the core levels of both metals (Figures S1–S3).^[^ ^26^, ^29^ ^]^ LEISS measurements for this system (Figure S4) indicated a Pd surface concentration of 21 ± 3%, further supporting Au surface segregation, in agreement with bulk PdAu alloy behavior.^[^ ^27^, ^30^ ^]^ Thermal desorption spectra (Figure S5a) revealed a decrease in the amount of adsorbed CO and a shift in CO desorption temperature from 480–500 K on Pd/CeO_2_(111) to 390 K on Pd_0.3_Au_0.7_/CeO_2_(111) (Figure S3), consistent with CO binding to perturbed Pd sites in the PdAu alloy.^[^ ^28^ ^]^ Thus, electronic interactions associated with Pd–Au bonding reduce the chemical reactivity of Pd,^[^ ^24^, ^26^, ^27^, ^28^ ^]^ as also confirmed by our theoretical calculations. DFT adsorption energies for CO decrease from −2.32 eV on Pd/CeO_2_ to −2.04 eV on Pd_20_/CeO_2_ and −1.75 eV on Pd_4_Au_16_/CeO_2_, consistent with the experimental trend (Figure S5b). Additionally, CO‐IRAS spectra collected at 100 K for CO adsorption on the Pd_0.3_Au_0.7_/CeO_2_(111) surface (Figure S6) exhibited a line shape that was very different from that of Pd/CeO_2_(111) or Au/CeO_2_(111) but was in very good agreement with IR spectra collected after adsorbing CO on Au‐rich bulk alloys.^[^ ^27^, ^28^ ^]^ The IR spectrum for CO bonded to Pd/CeO_2_(111) exhibited clear bands at 2110 and 1950 cm^−1^ (Figure S6), corresponding to CO bonded to atop and bridge sites of pure Pd particles.^[^ ^27^, ^28^, ^36^ ^]^ However, CO adsorption on the Pd_0.3_Au_0.7_/CeO_2_(111) displayed only the atop‐bonded Pd band at approximately 2090 cm^−1^ (Figure S6), indicating that the alloy catalyst primarily exposes isolated Pd atoms embedded within a Au matrix.^[^ ^27^, ^28^ ^]^ Importantly, these isolated Pd sites in the PdAu alloy differ from single Pd atoms on ceria, which are thermodynamically unstable and prone to aggregation under reaction conditions (see Figures S7 and S8).

### Preventing Methanol Decomposition Through Pd Alloying

The decomposition of methanol on Pd(111) has been extensively studied at both experimental^[^ ^23^, ^37^, ^38^, ^39^, ^40^, ^41^ ^]^ and theoretical^[^ ^42^, ^43^, ^44^, ^45^ ^]^ levels, as this surface serves as a benchmark for methanol synthesis and steam reforming. At very low temperatures (100–140 K), methanol adsorbs molecularly.^[^ ^38^, ^41^ ^]^ Upon heating to 180−250 K, the adsorbed CH_3_OH decomposes into H_2_ and CO via a *η* ^1^–C–*η* ^1^–O formaldehyde intermediate.^[^ ^37^, ^38^, ^41^ ^]^ A sequential decomposition pathway (CH_3_OH → CH_3_O → CH_2_O → CHO → C) has been proposed.^[^ ^37^, ^40^ ^]^ At 300 K, CO is the primary decomposition product when methanol is adsorbed on Pd(111) at 300 K.^[^ ^23^ ^]^ In contrast, methanol does not dissociate on Au(111).^[^ ^46^ ^]^ Instead, it adsorbs molecularly at 100 K and desorbs intact at temperatures below 200 K.

**Figure 1:**
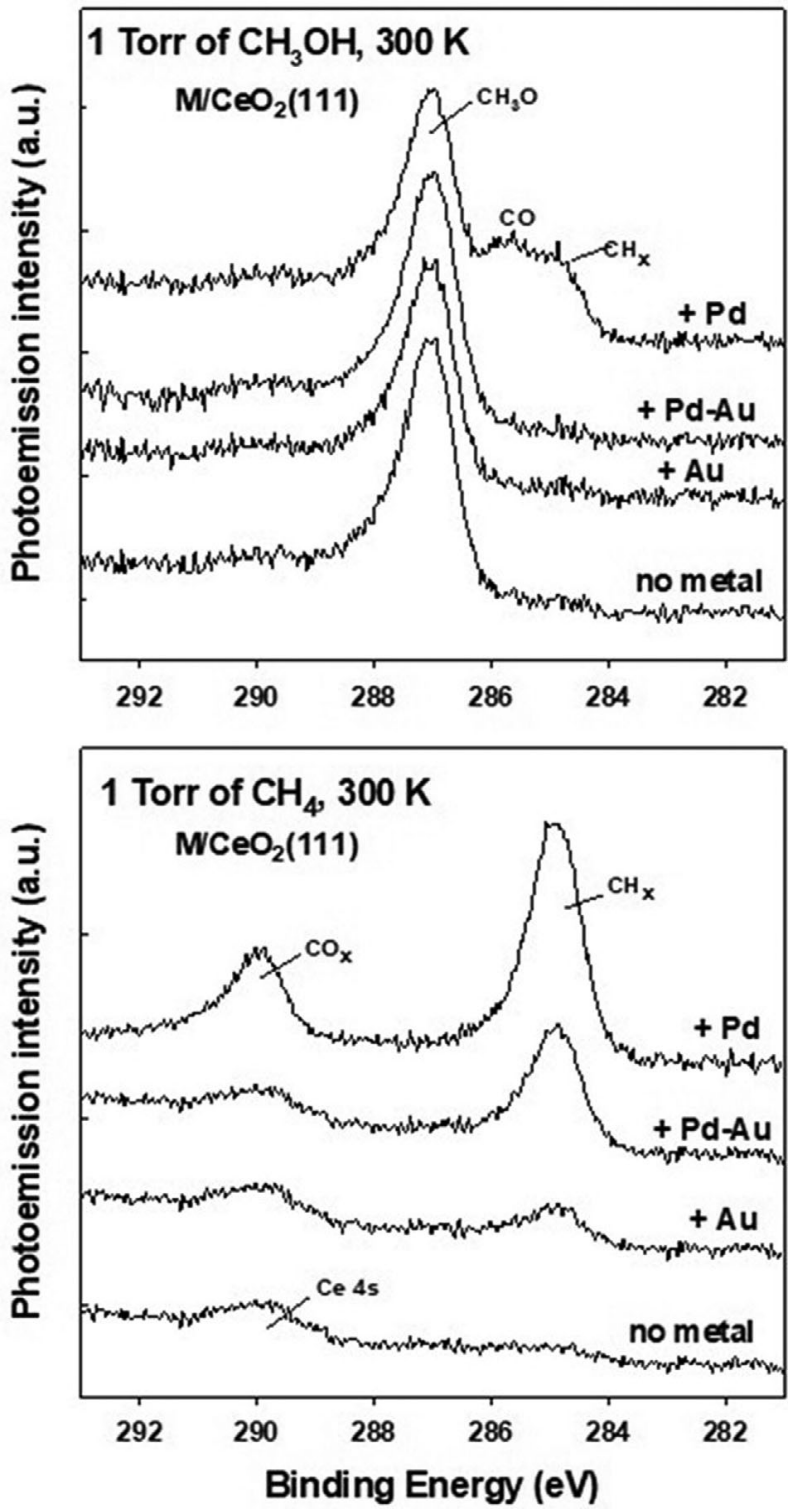
Dissociation of methanol (top) and methane (bottom) on different surfaces at room temperature. The Pd/CeO_2_(111) and Au/CeO_2_(111) systems contained 0.4 ML of the admetal. To generate the Pd_0.3_Au_0.7_/CeO_2_(111) system, 0.28 ML of Au was deposited onto a CeO_2_(111) surface pre‐covered with 0.12 ML of Pd.

The top panel in Figure 1 presents C 1s XPS spectra collected after exposing ceria surfaces pre‐covered with Au, Pd, and a Pd_0.3_Au_0.7_ alloy to 1 Torr of methanol at 300 K. On Pd/CeO_2_(111), methanol adsorption produces distinct spectral features corresponding to CO and CH*_x_* groups, consistent with previous findings.^[^ ^23^ ^]^ However, alloying Pd with Au eliminates this over‐reactivity, preventing CH_3_O dissociation on Pd_0.3_Au_0.7_/CeO_2_(111) (Figure 1) and plain Pd_0.3_Au_0.7_ (Figure S9). The C 1s peak at ∼287 eV for Pd_0.3_Au_0.7_/CeO_2_(111) matches that observed for methoxy groups on oxide surfaces^[^ ^21^, ^47^, ^48^, ^49^ ^]^ and Pd_0.3_Au_0.7_ (Figure S9). The moderate reactivity of these alloys may result from a reduced number of exposed Pd atoms,^[^ ^25^, ^26^, ^27^ ^]^ electronic effects induced by Pd–Au bimetallic bonding,^[^ ^24^, ^26^, ^29^ ^]^ or a combination of both. Figure S10 shows TPD spectra collected after heating the CH_3_O/Pd_0.3_Au_0.7_ and CH_3_O/Pd_0.3_Au_0.7_/CeO_2_(111) systems from 300 to 700 K. On the plain Pd_0.3_Au_0.7_, adsorbed methoxy underwent disproportionation (2CH_3_O → CH_2_O + CH_3_OH) to yield formaldehyde and methanol around 440 K. These features were also seen in the TPD spectra for CH_3_O/Pd_0.3_Au_0.7_/CeO_2_(111), and, in addition, there was desorption of CH_2_O and CH_3_OH coming from CeO_2_(111) around 560 K.^[^ ^47^ ^]^ Thus, the Pd_0.3_Au_0.7_/CeO_2_(111) composite displayed a chemical activity in between those seen for Au(111), no reaction with the alcohol, and Pd(111), extreme reactivity with extensive cleavage of C─O and C─H bonds.^[^ ^23^ ^]^ This alloy system meets a critical requirement, preventing methanol decomposition (reaction 5), which must be addressed before attempting synthesis of methanol via the CH_4_ + H_2_O → CH_3_OH + H_2_ process.

### Activation and Dissociation of Methane

Methane interacts very weakly with late transition metal surfaces, including Pd(111) and Au(111).^[^ ^11^ ^]^ In our studies, no evidence for methane dissociation was observed on either Pd(111) or Pd_0.3_Au_0.7_ surfaces (bottom panel, Figure S11). However, metal‐oxide interactions can significantly enhance the reactivity of metals toward methane.^[^ ^50^ ^]^ For example, the activation barrier for C─H bond cleavage is notably lower on Pd/CeO_2_(111) compared to Pd(111),^[^ ^9^ ^]^ leading to methane dissociation on supported Pd (bottom panel, Figure 1). The reaction CH_4_ → (4−*x*)H + CH*_x_* primarily results in the formation of CH_2_ and CH_3_ fragments (∼285 eV peak).^[^ ^51^ ^]^ However, a substantial fraction of methane undergoes complete decomposition, forming C atoms that react with O centers to generate carbonate‐like species (∼290 eV feature).^[^ ^40^, ^52^ ^]^ The same Pd–CeO_2_ interface sites responsible for methanol and methoxy dissociation (top panel, Figure 1) likely contribute to CH*_x_* decomposition. Interestingly, depositing a Pd–Au alloy onto the ceria substrate significantly moderates this reactivity, and full decomposition into CO*_x_* is negligible. (The features near ∼290 eV likely originate from the Ce 4s core level of the CeO_2_(111) substrate). On Pd_0.3_Au_0.7_/CeO_2_(111), the coverage of CH*_x_* species resulting from methane decomposition is substantially lower than on Pd/CeO_2_(111). However, in the alloy/oxide composite, this reduction in chemical activity is offset by the elimination of sites that would otherwise degrade the CH_3_O intermediate or methanol.

**Figure 2:**
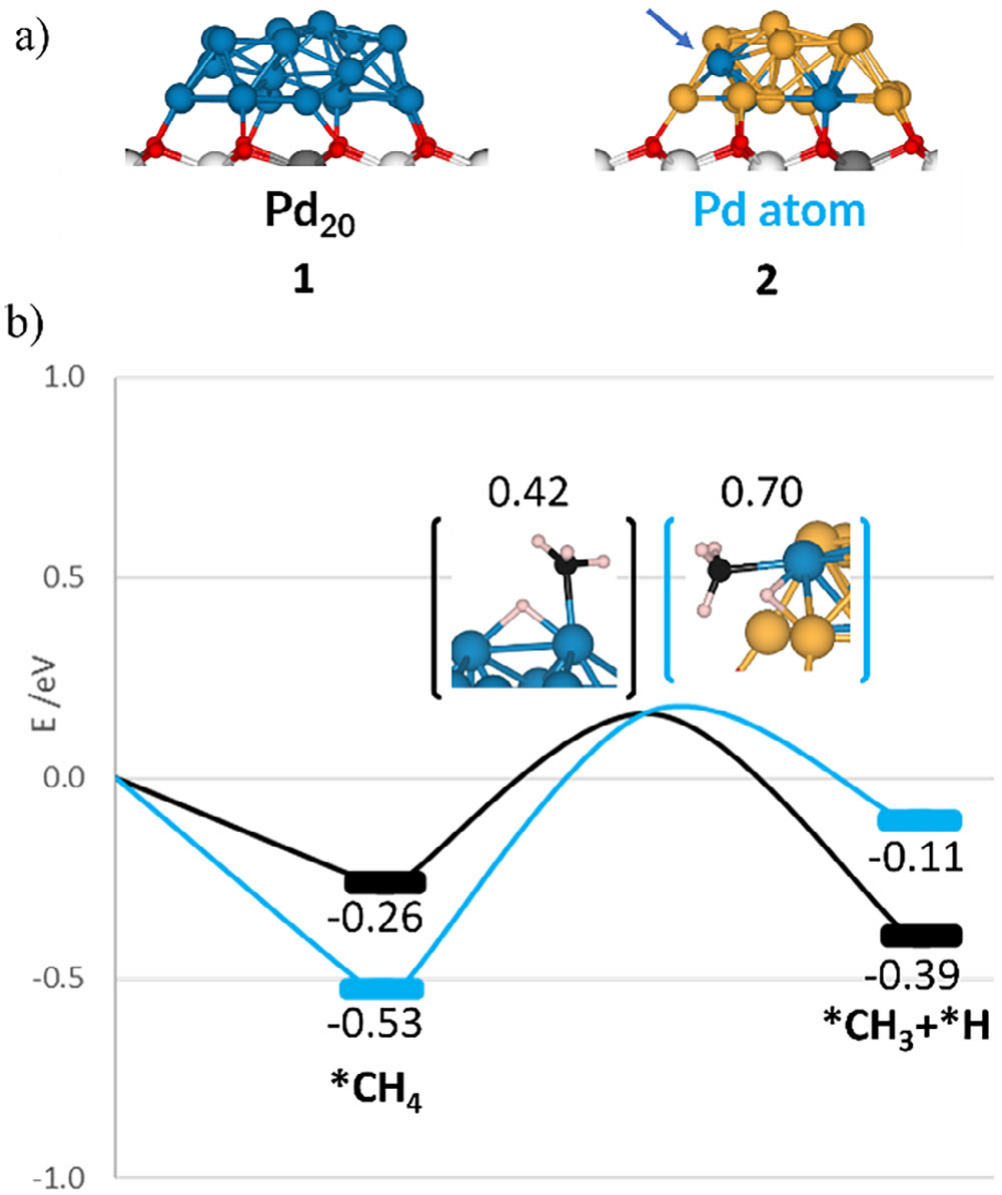
a) DFT‐optimized Pd_20_/CeO_2_(111) and Pd_4_Au_16_/CeO_2_(111) models, representing the Pd/CeO_2_(111) and Pd_0.3_Au_0.7_/CeO_2_(111) experimental catalysts, respectively (see also Figure S1). The blue arrow indicates the Pd active site in Pd atom model. b) Energy profile for DFT‐calculated CH_4_ dissociation on Pd_20_/CeO_2_ (black) and on Pd_4_Au_16_/CeO_2_ (light blue). Energies (in eV) are referenced to each model + CH_4_(gas). Activation energies are indicated above the transition state structures, with close‐up views shown in parentheses. Full structures are provided in Figure S17. All figures use the following color scheme: Ce⁴⁺ (white), Ce^3^⁺ (gray), O (red), Pd (blue), Au (gold), C (black), and H (white). Asterisks (*) denote chemisorbed species.

To gain deeper insight into the mechanism of methane dissociation on Pd/CeO_2_(111) and the PdAu/CeO_2_(111) composite catalyst, DFT calculations were performed. Structural modeling of metallic and bimetallic clusters on the CeO_2_(111) surface was carried out using the Global Optimization with First‐Principles Energy Expression (GOFEE) method.^[^ ^53^ ^]^ Two catalyst models were considered: a Pd_4_Au₁₆ cluster and a Pd_2_₀ cluster, both supported on a flat CeO_2_(111) surface (Figure 2a). A detailed exploration of their possible geometries on the oxide substrate was performed within this framework, with additional details available in the Supporting Information (Figures S12–S15 and Tables S1–S3).

For the Pd_4_Au_16_/CeO_2_(111) catalyst model, calculations identified the most stable configuration as one in which three Pd atoms are encapsulated by Au, leaving a single Pd atom exposed. This structure, hereafter referred to interchangeably as the “Pd atom” or Pd_4_Au_16_ model, aligns well with experimental observations from CO‐IRAS and LEISS, which indicate a high concentration of Au and isolated Pd atoms on the surface of the metal particles. To compare the behavior of the Pd atom model and the Pd_20_ cluster in methane activation, CH_4_ adsorption was systematically studied on both models, exploring various bonding configurations (Figure S16) to assess their potential for dissociation. The site with the lowest barrier for the CH_4_ → CH_3 _+ H reaction (Figures 2 and S17) is the exposed six‐fold coordinated Pd site adjacent to Au in the Pd_4_Au_16_ cluster, which exhibits an adsorption energy of ∆*E*_ads_ = −0.53 eV (Figure S17, structure **S51**). This value is nearly twice the adsorption energy observed on a seven‐fold coordinated Pd site in the Pd_20_ cluster (cf. −0.26 eV, Figure S17, structure **S49**).

Previous studies^[^ ^9^, ^50^ ^]^ have shown that low metal loading and the presence of the ceria support play a crucial role in lowering activation barriers for CH_4_ dissociation on small Ni, Co, Pd, and Pt clusters in direct contact with a CeO_2_(111) support. In these M/CeO_2_ systems (M = Ni, Co, Pd, Pt), oxidized metal sites (M^δ+^) at the metal‐ceria interface enhance CH_4_ pre‐activation via a ligand effect. ^[^ ^9^, ^50^ ^]^ This interaction allows CH_4_ to adsorb closer to the surface, where a C─H bond is weakened, facilitating its cleavage more effectively than on the corresponding extended metal surfaces.

In the ceria‐supported Pd_4_Au₁₆ and Pd_2_₀ clusters, the formation of four and three Ce^3^⁺ sites, respectively, indicates that the CeO_2_ support influences both systems. Bader charge analysis reveals distinct electronic behaviors for the Pd sites in the two models (Figure S12 and Table S1). In both cases, metal–support interactions lead to partial oxidation of Pd atoms in direct contact with CeO_2_, as seen in previous studies,^[^ ^35^ ^]^ resulting in small positive charges that do not reach formal +1 or +2 oxidation states. This clearly shows that the support modifies the electronic properties of the Pd sites, regardless of alloying.

In the Pd_4_Au₁₆/CeO_2_ model, alloying with Au introduces an additional effect. The charge redistribution within the PdAu nanoparticle arises from the combined influence of alloying and support interactions. The exposed isolated Pd atom in Pd_4_Au₁₆/CeO_2_ exhibits a charge deficit (+0.08 |e^−^|), consistent with partial oxidation (Pd^δ+^) and the large electronegativity of Au.^[^ ^24^, ^29^ ^]^ In contrast, the active Pd site in Pd_2_₀/CeO_2_ remains metallic (Pd^0^). As for the active site for methane adsorption, in Pd_20_, the active Pd site is metallic (Pd^0^), whereas in the Pd atom model, the exposed Pd site exhibits a charge deficit (+0.08 |e^−^|), consistent with partial oxidation (Pd^δ+^) and the large electronegativity of Au.^[^ ^24^, ^29^ ^]^ The dilution of surface Pd by Au is further reflected in a relative shift in the d‐band center of the projected d‐density of states on the Pd adsorption sites in both systems (Figure S18). These combined effects significantly influence the reactivity of Pd sites in Pd/CeO_2_ and PdAu/CeO_2_ compared to their unsupported counterparts, Pd(111) and PdAu(111), particularly in their interactions with methane and water.

Consistent with the previous findings,^[^ ^9^, ^50^ ^]^ in the chemisorbed state on the isolated Pd^δ+^ site in the Pd_4_Au_16_ cluster, CH_4_ absorbs 10 pm closer to the surface and exhibits a 0.5 pm greater C─H bond elongation compared to adsorption on the Pd^0^ site in the Pd_20_ cluster (Figure S16). A detailed analysis of the adsorption energies of CH_3_ and H—and their impact on reaction energetics—is provided in the Supporting Information (Figures S17 and S19).

These results align remarkably well with the experimental findings in Figure 1. The bimetallic model exhibits an activation barrier that can be overcome under the mild experimental conditions, consistent with the presence of CH*_x_* peaks in the XPS spectra, while the high endothermicity prevents further dehydrogenation. In contrast, the significantly low barrier and high exothermicity of the Pd_20_/CeO_2_ model fully support the stepwise dehydrogenation of CH*_x_* species and their subsequent oxidation to CO*_x_*, as observed experimentally in Figure 1.

### Activation and Dissociation of Water

Water is the second reactant required for methanol synthesis via reaction (1). The XPS results in Figure S20 indicate that water does not dissociate on Pd(111) or Pd_0.3_Au_0.7_(111) surfaces, consistent with previous reports for Au(111).^[^ ^54^ ^]^ Theoretical studies have also shown that water dissociation on Pd(111) or Au(111) is energetically unfavorable.^[^ ^55^, ^56^ ^]^ In contrast, ceria can facilitate water dissociation^[^ ^57^ ^]^ but the rate of the H_2_O_gas_ → OH_ads_ + H_ads_ process is substantially enhanced by the presence of supported metal atoms.^[^ ^58^ ^]^ The XPS results in Figure S20 confirm that the Pd_0.3_Au_0.7_/CeO_2_(111) composite interacts effectively with water and the molecule dissociates to deposit OH groups on the surface at 300 and 500 K. This reactivity may be attributed to the presence of corner atoms in supported metal nanoparticles^[^ ^54^, ^55^ ^]^ and the formation of a reactive metal‐oxide interface,^[^ ^54^ ^]^ both of which can facilitate O─H bond cleavage.

As with methane activation, DFT calculations were performed to investigate water dissociation on PdAu/CeO_2_. First, H_2_O adsorption was examined on the Pd site of the Pd atom model. The water molecule adsorbs at the metal–ceria interface, interacting with both the metal and the ceria surface (Figure S21). This interaction leads to an adsorption energy approximately twice as strong as that of CH_4_ at the same Pd site (cf. Figures 2 and S21).

Since water molecules adsorb at the interface, they form hydrogen bonds with surface O atoms (O_s_) and subsequently dissociate, producing an *O_s_H species (Figure S21). This dissociation process requires an interfacial Au atom to weaken its interaction with a surface oxygen atom and to shift upwards, allowing the previously bonded O_s_ to accept the hydrogen. As a result, water dissociation becomes an endothermic process, yet it still proceeds with an affordable barrier of 0.69 eV (Figure S21).

These findings demonstrate that water adsorption and dissociation occur at the Pd–ceria interface, aligning well with the experimental trends observed in Figure S20. Therefore, the preliminary XPS and DFT studies presented in Figures 1, 2, S20, and S21 indicate that the Pd_0.3_Au_0.7_/CeO_2_(111) composite possesses the ideal properties to catalyze the reaction between methane and water, ultimately leading to methanol formation.

### Reaction of Methane with Water: Methanol Formation (Catalytic Tests)

**Figure 3:**
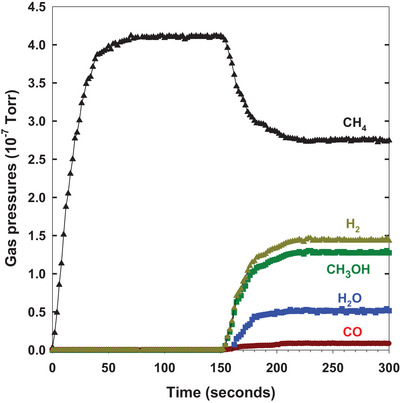
Pulse study conducted on a pristine Pd_0.3_Au_0.7_/CeO_2_(111) surface at 500 K. At *t* = 0 s, 4 × 10^−7^ Torr of methane was introduced into the chamber containing the catalyst. At *t* = 150 s, water was added at an equivalent pressure of 2 × 10^−7^ Torr, with most water reacting with methane to yield methanol.

Figure 3 presents a pulse study examining the reaction of CH_4_ with H_2_O on the Pd_0.3_Au_0.7_/CeO_2_(111) surface at 500 K. In the UHV chamber used for the preparation of the composite, a mass spectrometer was positioned near the Pd_0.3_Au_0.7_/CeO_2_(111) surface, and sequential pulses of methane (4 × 10^−7^ Torr) and water (2 × 10^−7^ Torr) were introduced. The addition of water led to the formation of methanol (1) and CO (2), with no CO_2_ detected. H_2_ could be produced by reactions (1) and (2). Approximately 75% of the introduced water reacted with methane. Under steady‐state conditions, the selectivity for methanol production reached 90%.

**Figure 4:**
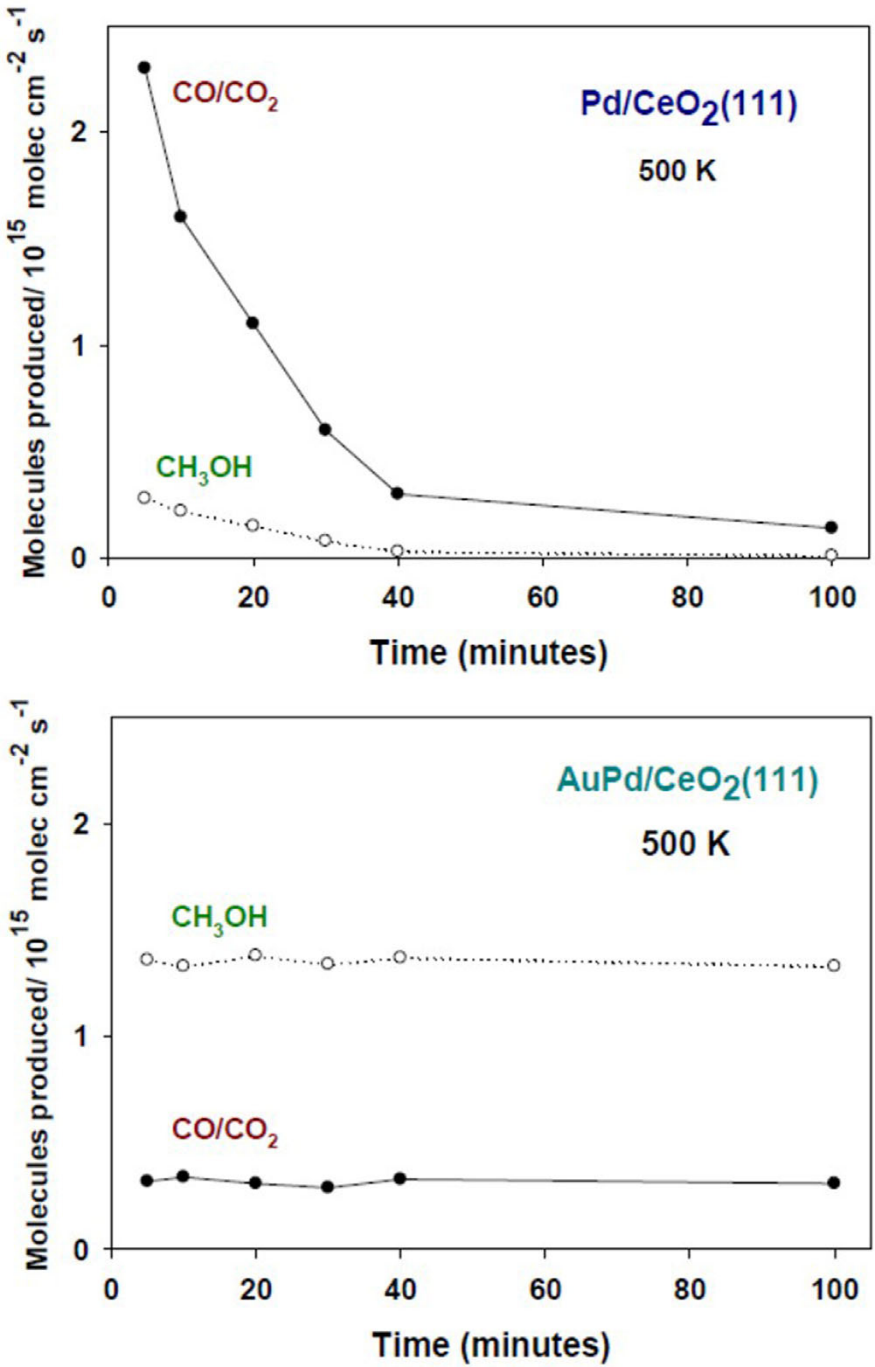
Conversion of methane (1 Torr) by reaction with water (1 Torr) at 500 K over Pd/CeO_2_(111) (top) and Pd_0.3_Au_0.7_/CeO_2_(111) (bottom). Methanol is formed via reaction (1), while CO and CO_2_ originate from reactions (2) and (3). Both systems contained 0.12 ML of Pd. The alloy was generated following the same methodology used for the experiments in Figures S2 and S3: 0.28 ML of Au were deposited on the Pd/CeO_2_(111) surface at 300 K with subsequent annealing at 600 K.

Using a batch reactor, we investigated the CH_4_ + H_2_O → CH_3_OH + H_2_ reaction on Pd/CeO_2_(111) and Pd_0.3_Au_0.7_/CeO_2_(111) surfaces at 500 K (Figure 4). On Pd/CeO_2_(111), the main reaction products were CO and CO_2_, resulting from reactions (2) and (3). In contrast, methanol production was almost 10 times smaller. Moreover, the Pd/CeO_2_(111) catalyst was not stable and eventually deactivated due to carbon deposition from the full decomposition of methane. This accumulated carbon likely blocked the active sites responsible for water dissociation, further hindering catalytic activity. In contrast, the Pd_0.3_Au_0.7_/CeO_2_(111) catalyst exhibited remarkable stability, with methanol as the dominant product. From the data displayed at the bottom of Figure 4, and assuming that the chemistry takes place on Pd sites, we estimate a turnover number for the production of methanol in the range of 2–4 molecules per second. The selectivity for methanol production was approximately 80%, making this PdAu/CeO_2_ composite superior to other systems capable of a direct CH_4_ + H_2_O → CH_3_OH + H_2_ conversion.^[^ ^12^, ^13^, ^20^, ^21^ ^]^

It must be stressed that the catalytic activity seen in Figure 3 and the bottom of Figure 4 is a direct consequence of the formation of a PdAu alloy. Experiments with small coverages (0.02–0.12 ML) of Pd on ceria under similar catalytic conditions did not yield any significant amount of methanol (Figure S22). These systems were too reactive toward methane and postreaction characterization with XPS showed a combination of CH*_x_* and C on the surface of both catalysts (Figure S23). The accumulation of C/CH*_x_* is problematic because it eventually deactivates Pd centers in plain Pd(111) and powder catalysts.^[^ ^23^, ^37^, ^40^ ^]^ The high reactivity of our Pd/CeO_2_ systems toward CH_4_ is consistent with the fact that single atoms of Pd embedded in ceria are very good catalysts for the full destruction and oxidation of methane.^[^ ^59^ ^]^ For the Pd_0.3_Au_0.7_/CeO_2_(111) catalyst in Figure 4, postreaction characterization with XPS showed the presence of CH_3_O and OH groups on the surface (Figure S23 and S24), with no signs of a reduction of the ceria support (Figure S24). The line shape of the Ce 3d spectrum matches that reported for stoichiometric CeO_2_.^[^ ^52^ ^]^ The contrast seen in the C 1s spectra of the Pd/CeO_2_(111) and Pd_0.3_Au_0.7_/CeO_2_(111) catalysts after reaction (Figure S23) reflects the fact that the alloy does not destroy the CH_3_O and CH_3_OH groups (Figures 1 and S9).

As shown in Figures 4 and S25, the Pd_0.3_Au_0.7_/CeO_2_(111) catalyst did not show any signs of deactivation after a long period of operation or several cycles. Figure S26 summarizes experiments in which the Au concentration in Pd_1‐_ *_x_* Au*_x_*/CeO_2_(111) was systematically varied. The two extremes correspond to Pd/CeO_2_(111) and Au‐rich Pd_1‐_ *_x_* Au*_x_*/CeO_2_(111) surfaces. For pure Au/CeO_2_(111), methane dissociation is negligible (Figure 1, bottom panel), confirming that Au alone is not an active catalyst for methanol production. Figure S26 shows that increasing the Au content in Pd_1‐_ *_x_* Au*_x_*/CeO_2_(111) results in a monotonic decrease in methane conversion, while methanol selectivity increases once the Au concentration exceeds 50%, eventually reaching a plateau between 70 and 90%. The Pd_0.3_Au_0.7_/CeO_2_(111) composite represents an optimal balance between conversion and selectivity.

According to reactions (2) and (3), the methane‐to‐water ratio is a critical parameter. At a 1:1 ratio, methane conversion to CO is highly endothermic, allowing methanol production (reaction (1)) to compete. However, at a 1:2 ratio, the favored reaction can shift toward CO_2_ formation. Figure S27 illustrates the impact of water concentration on methanol selectivity for the Pd_0.3_Au_0.7_/CeO_2_(111) catalyst. At 1:2 and 1:3 ratios, surface sites became saturated with OH groups, reducing CH_4_ conversion by 60%−85% compared to the 1:1 methane‐to‐water ratio (Figure S26, bottom panel). Additionally, a selectivity shift toward CO/CO_2_ production, further diminishing methanol yield. Pulse experiments similar to those in Figure 3 were done in which a *t* = 0 a pressure of 4 × 10^−7^ Torr of water was introduced into the chamber containing the catalyst. At *t* = 150 s, methane was added at an equivalent pressure of 2 × 10^−7^ Torr. Under this water rich environment, the rate of methanol formation was low, and CO and CO_2_ were dominant reaction products.

### Reaction of Methane with Water: Mechanism for Methanol Formation (DFT Studies)

**Figure 5:**
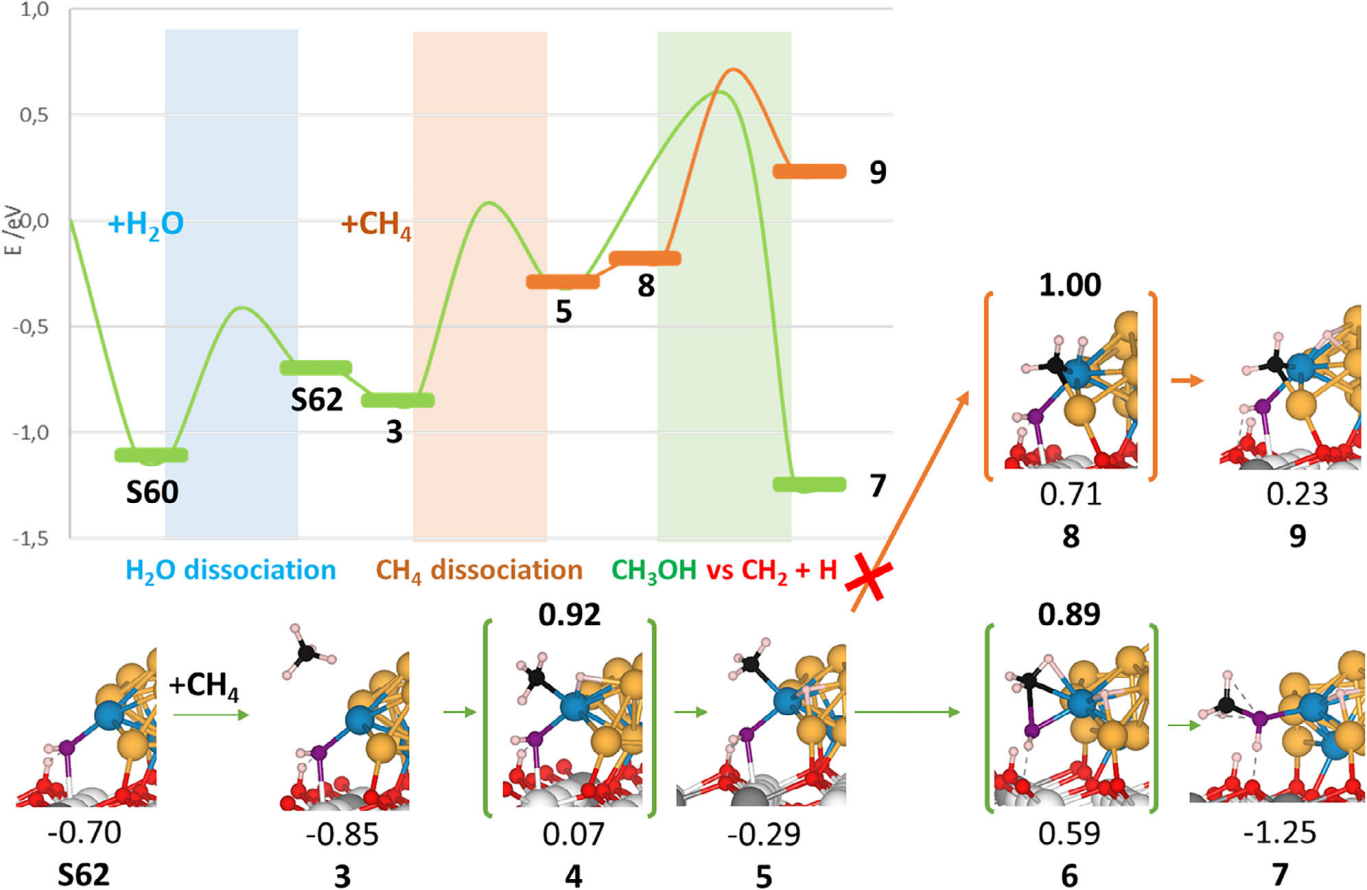
Energy profile for the DFT calculated H_2_O + CH_4_ reaction to methanol on the Pd atom (green) model, along with the competitive CH_3_ dissociation pathway (orange). Close‐up structures are shown (see Figures S29 and S30 for complete structures). Energies (in eV) are referenced to the Pd atom model + CH_4_(gas) + H_2_O(gas). Activation energies are indicated above the transition state structures. Color scheme: same as Figure 2, with O from H_2_O shown in purple.

Since water adsorbs more strongly than methane at the PdAu–CeO_2_ interface (by ∼0.6 eV) and its dissociation is energetically feasible (activation barrier of 0.69 eV, comparable to 0.70 eV for CH_4_ activation in the absence of OH), we investigated the full CH_4_ + H_2_O reaction starting from the *OH + *OsH configuration (structure **S62** in Figure S21). This choice reflects a chemically realistic and mechanistically relevant scenario in which water dissociation precedes methane activation. Moreover, prior studies^[^ ^58^ ^]^ demonstrated the crucial role of the metal–ceria interface in enhancing water binding and promoting O─H bond cleavage, further supporting the relevance of starting from pre‐formed OH species in our analysis. Even after water dissociation, the active Pd atom site in the Pd atom model remains exposed, now with an *OH species adsorbed. As a result, CH_4_ adsorption still occurs on that same site (Figure 5). However, compared to adsorption in the absence of water, the presence of OH species negatively affects methane adsorption. The CH_4_ adsorption energy decreases from −0.53 eV (Figures 2, structure **S51** and S16b) to −0.15 eV (structure **3**, Figures 5 and S28a), and the C─H bond activation is correspondingly weaker, with bond lengths of 1.125 and 1.100 Å, respectively.

Following methane adsorption, the first C─H bond dissociation occurs, leading to the formation of *CH_3_ and *H species, both absorbed on the same Pd atom (structure **5**, Figure 5). This step is identified as the first rate‐limiting step in the reaction pathway, with an activation barrier of 0.92 eV (transition state structure **4**, Figure 5). The dissociated *H species remains at the Pd site, alongside with the adsorbed *CH_3_ group. Notably, the activation barrier increases by only 0.22 eV, and the reaction energy by 0.14 eV compared to the case without water (cf. Figures 2 and 5). While this increase is expected, it remains moderate, considering the temperature and pressure conditions experimentally studied for this reaction in Figures 3 and 4.

The next step involves the reaction between *CH_3_ and *OH species to form methanol. This step has a slightly lower activation barrier of 0.89 eV (structure **6**, Figure 5), making it comparable in energy requirements to the first dissociation step. During this process, the Pd atom facilitates the recombination of *CH_3_ and *OH, leading to the formation of *CH_3_OH, which subsequently desorbs from the surface as the final product.

Notably, competing side reactions, such as the further dissociation of *CH_3_ into *CH_2_ and *H, are suppressed in the Pd atom model (Figure 5). This effect arises from the limited availability of Pd active sites and the destabilizing influence of the surrounding Au atoms on the dissociated *CH_2_ + *H species. The energy barrier for *CH_3_ dissociation requires 1.0 eV (structure **8**, Figures 5 and S30), making this pathway energetically less favorable than methanol formation. Even more significant than the activation barrier is the endothermicity of this process (∼0.5 eV), regardless of whether the final structure involves a Pd–Au (or Au–Au) edge (Figure S31). This contrasts sharply with the exothermicity of approximately 1.0 eV observed for methanol formation, further disfavoring CH_3_ dissociation. These findings confirm the study's hypothesis that Au inclusion effectively stabilizes *CH_3_ species, promoting methanol formation while preventing undesired side reactions.

The reaction mechanism for methane‐to‐methanol conversion on the Pd atom model involves two key steps: CH_4_ dissociation into *CH_3_ and *H, followed by the recombination of *CH_3_ and *OH to form methanol. Both steps are rate‐determining with activation barriers of approximately 0.9 eV. The presence of Au atoms plays a crucial role in modulating Pd activity, preventing the over‐dissociation of intermediates, and enhancing methanol selectivity. Additionally, the CeO_2_ support stabilizes the active Pd sites and facilitates water dissociation, ensuring a continuous supply of *OH groups necessary for the reaction. This model is representative of low Pd:Au ratio systems and thus aligns fully with the experimental findings for the Pd_0.3_Au_0.7_/CeO_2_(111) catalyst (Figures 3 and 4). Previous studies have shown that the chemical properties of Pd can significantly be modified by alloying,^[^ ^16^, ^17^, ^18^, ^24^, ^25^, ^26^, ^27^, ^28^, ^60^, ^61^ ^]^ and that Pd single‐site catalysts can exhibit a performance markedly different from that of bulk‐like Pd particles.^[^ ^61^, ^62^, ^63^ ^]^ In the PdAu/CeO_2_ composite, metal–metal and metal–oxide interactions combine synergistically to control both activity and selectivity, making this catalyst system highly effective for selective methane conversion to methanol.

## Conclusion

This study demonstrates the exceptional performance of PdAu/CeO_2_ composite catalysts for the selective and efficient conversion of methane and water into methanol under mild conditions, offering critical insights into alloying effects and metal–oxide interface engineering. Experimental and computational results revealed that the Pd_0.3_Au_0.7_/CeO_2_ composite achieves remarkable selectivity (∼80% at a 1:1 methane‐to‐water ratio) and superior stability, significantly outperforming Pd/CeO_2_ in methanol production.

The strategic alloying of Pd with Au plays a pivotal role in tuning Pd reactivity, mitigating over‐dissociation of intermediates, and preserving high selectivity by reducing the number of highly reactive Pd sites. The synergy between Pd, Au, and CeO_2_ is most evident at the Pd_0.3_Au_0.7_/CeO_2_ interface, where partially oxidized isolated Pd atoms, stabilized by both alloying and support interactions, enable methane activation while effectively suppressing CO and CO_2_ formation.

DFT calculations further confirm that isolated Pd atoms at the PdAu–CeO_2_ interface are key to balancing activity and selectivity. Additionally, the CeO_2_ support plays a critical role in promoting water adsorption and dissociation, ensuring a continuous supply of *OH groups necessary for a selective CH_4_ + H_2_O → CH_3_OH reaction.

These findings underscore the importance of precise control over alloy/oxide interfaces in catalyst design and establish the Pd_0.3_Au_0.7_/CeO_2_ composite as a highly promising candidate for methane valorization. This work paves the way for the development of next‐generation catalytic systems with superior efficiency, selectivity, and long‐term stability in C1 chemistry.

## Conflict of Interests

The authors declare no conflict of interest.

## Supporting information



Supporting Information

## Data Availability

The data that support the findings of this study are openly available in Materials Cloud at 10.XXX/materialscloud:XXXX, reference number [REF].
