# Supplementary material for: Engineering PdAu/CeO2 Alloy/Oxide Interfaces for Selective Methane‐to‐Methanol Conversion with Water
Source: Angew Chem Int Ed Engl. 2025 Jul 24;64(36):e202505716. doi: 10.1002/anie.202505716 (PMC12402882; doi:10.1002/anie.202505716)
Supplement: Supplementary file 1 — Supporting Information [file ANIE-64-e202505716-s001.pdf]

Engineering PdAu/CeO_2 Alloy/Oxide Interfaces for Selective Methane-to-Methanol Conversion with Water

Estefanía Fernández-Villanueva,[1,2,3] Pedro J. Ramírez,[4,5] Pablo G. Lustemberg,[2,*] Rubén Pérez,[3,6] M. Verónica Ganduglia-Pirovano,[2,*] and José A. Rodriguez[7,*]

[1] *Universitat Politècnica de València, Camí de Vera s/n, 46022 Valencia, Spain*

[2] *Instituto de Catálisis y Petroleoquímica (ICP-CSIC), C/ de Marie Curie 2, 28049 Madrid, Spain*

[3] *Departamento de Física Teórica de la Materia Condensada, Universidad Autónoma de Madrid, E-28049 Madrid, Spain*

[4] *Facultad de Ciencias, Universidad Central de Venezuela, Caracas 1020-A, Venezuela*

[5] *Zoneca-CENEX, R&D Laboratories, Alta Vista, 64770 Monterrey, México*

[6] *Condensed Matter Physics Center (IFIMAC), Universidad Autónoma de Madrid, E-28049, Madrid, Spain*

[7] *Chemistry Division, Brookhaven National Laboratory, Upton, New York 11973, United States*

*Corresponding authors: Pablo G. Lustemberg, p.lustemberg@csic.es; M. Verónica Ganduglia-Pirovano, vgp@icp.csic.es; José A Rodriguez, rodrigez@bnl.gov

Supporting Information

Experimental and theoretical methods ………………….... S2

Results ……………………………………………..…….. S6

Experimental and theoretical methods:

A. Preparation and characterization of PdAu and PdAu/CeO_2(111) surfaces

Bulk alloys of PdAu with varying compositions (from Pd-rich to Pd-poor) were prepared by depositing Pd and Au atoms onto a Mo(110) surface at 300 K, followed by annealing to 600−700 K.[27] To obtain a $Pd_{0.3}Au_{0.7}$ system, 2.2 ML of Pd and 5 ML of Au were deposited and intermixed on the Mo(110) surface. Core and valence photoemission spectroscopy were used to verify the intermixing of Pd and Au.[27,29] The Pd concentration on the alloy surfaces was determined using He^+ low-energy ion-scattering spectroscopy (LEISS),[27,30] along with CO chemisorption at room temperature, temperature programmed desorption (TPD),[28,25] and Fourier-transform infrared reflection absorption spectroscopy (FT-IRAS).[24,25,27] To generate PdAu alloys on a CeO_2(111) support, sequential deposition of Pd and Au was employed. First a 30−40 Å thick CeO_2(111) film was growth on a Ru(0001) substrate.[34,64] Then, Pd was deposited first onto the ceria to create nucleation centers for Au.[8] To form the $Pd_{0.3}Au_{0.7}/CeO_2$(111) composite, 0.28 ML of Au were deposited onto a CeO_2(111) surface pre-covered with 0.12 ML of Pd. The resulting PdAu/CeO_2(111) composites were then annealed at 600 K, and their alloy properties were examined using photoemission, LEISS, CO-TPD and CO-IRAS.[24,25,27-30]

The chemical and catalytic properties of the PdAu and PdAu/CeO_2(111) surfaces were investigated in a set-up that combined a ultra-high vacuum (UHV) chamber for surface characterization with a batch microreactor.[20,21,54] The sample could be transferred between the two components of this system without air exposure. The UHV chamber was equipped with instrumentation for X-ray photoelectron spectroscopy (XPS; Mg Kα source), LEISS and TPD.[20,21,54] In the batch microreactor, the different samples were exposed to 1 Torr of CH_4, CH_3OH or H_2O at 300 K for 5 minutes. After exposure, the gases were evacuated, and the samples were transferred to the UHV chamber for surface characterization via XPS. In the catalytic tests,

fresh samples were exposed to 1 Torr of CH_4 and 1 Torr of H_2O at 300 K, followed by a temperature ramp to 500 K, where the measurements were performed. The formation of methanol, CO, and CO_2 was monitored by a combination of gas chromatography and mass spectroscopy.[21,54,65] Kinetic studies were conducted always under low methane conversion (<5%). The steady-state for the $Pd_{0.3}Au_{0.7}/CeO_2(111)$ composite was reached after 2–3 min of reaction. Under CH_4/H_2O mixtures, no signs of catalyst deactivation were observed over 100 minutes of continuous operation.

B. Modeling and DFT calculations

To determine a reasonable model for the Pd_{20} and Pd_4Au_{16} supported clusters, we used the machine-learning-assisted "global optimization with first principles energy expression (GOFEE)"[53] method to efficiently explore a larger number of possible structures and at a lower computational cost than the density functional theory (DFT) calculations alone. For the single-point DFT calculations triggered by GOFEE, we employed the VASP code (version 5.4.4)[66-69] with a low precision preset. The projector augmented wave (PAW) method[70] was used to describe the valence electrons of the atomic species (Ce (4f, 5d, 6s), O (2s, 2p), Pd (4p, 4d, 5s), Au (5d, 6s), C (2s, 2p), and H (1s)), with a plane-wave cutoff energy of 415 eV. The generalized gradient approximation (GGA) of Perdew, Burke, and Ernzerhof (PBE)[71] was applied. Only the Γ-point was used to sample the reciprocal space, and no DFT+U or dispersion corrections were included at this stage. Following the schemes reported by Quinlivan et al. [72], we performed multiple GOFEE runs to mitigate the intrinsic initial structure bias. Each GOFEE run consisted of 400 cycles, generating more than 800 structures per run, evaluated through single-point calculations. In each cycle, 20 new candidates were generated, and the initial population size was 30, leading to the relaxation of thousands of structures using the surrogate model. The probability

rules for generating new candidates were set as follows: 60% from random structures, 20% from rattling, and 20% from element permutation. The final scheme involved three GOFEE runs, and the most stable unique structures from each run (within 2 eV) were fully optimized with DFT at a higher precision preset. This included a plane-wave cutoff energy of 415 eV, a Monkhorst−Pack (2 × 2 × 1) k-point mesh, the DFT+U approach (Dudarev et al.[73]) with a U_{eff} value of 4.5 eV for the Ce 4f electrons, and long-range dispersion interactions using the DFT-D3 Grimme correction.[74,75]

The $CeO_2(111)$ surface was modeled with (4 × 4) periodicity, an optimized lattice constant of 5.485 Å, and two O−Ce−O trilayers, separated by approximately 30 Å of vacuum. During GOFEE runs, the entire ceria support was fixed, while during VASP geometry optimizations, only the bottom O−Ce−O trilayer was fixed at optimized bulk-truncated positions, allowing all other atoms to relax. In the first GOFEE run, only one O−Ce−O trilayer of ceria was modelled, whereas the second and third runs included two trilayers, as used in the final models. Since VASP optimizations indicated that cluster deposition onto the $CeO_2(111)$ surface typically led to the formation of two Ce^{3+} ions due to cluster oxidation, two Ce^{3+} sites were explicitly included in the final GOFEE run. For Pd_{20}, the most stable structure identified up to that point was **S2** (Figure S5), with Ce^{3+} ions positioned at sites 6 and 16 (see Scheme S1 below), while for Pd_4Au_{16} the most stable structure was **S23** (Figure S6), with Ce^{3+} ions at sites 4 and 6. These Ce^{3+} positions where included and kept fixed in the final GOFEE run for the respective clusters.

After the final GOFEE runs (with these two Ce^{3+} positions fixed) and subsequent VASP optimizations (without constraints on Ce^{3+} sites), structures **S1** and **S17** emerged as the most stable ones. However, while investigating the reaction mechanism, we identified a more stable

configuration for both the number and placement of Ce^{3+} ions, leading to the final reported structures **1** and **2** (Figure 3 and S4).

For each supported cluster (CL) model, the adsorption energy of each molecule (ΔE_{ads}) was calculated as:

$$\text{A.}\quad \Delta E_{ads} = E(\text{Molecule/Slab}) - E(\text{Molecule}) - E(\text{Slab})$$

where E(Molecule/Slab) is the total energy of the CL_{20}/CeO_2 surface with an adsorbed molecule, E(Slab) is the energy of the bare CL_{20}/CeO_2 surface, and E(Molecule) is the energy of the gas-phase molecule. Isolated molecules were optimized at the higher precision preset in a 15 × 15 × 15 $Å^3$ cubic box with Γ k-point sampling of the Brillouin zone.

To identify transition state (TS) structures, we applied the climbing image nudged elastic band (CI-NEB)[76] method or the improved DIMER[77,78] method. All TS structures exhibited a single imaginary frequency. Within the computed potential energy profiles, the activation energy (ΔE_{act}) is defined as the difference between the energy of the transition state (TS) and the initial state (IS). The reaction energy (ΔE_{reac}) is defined as the difference between the final state (FS) and the initial state (IS).

Atomic charges were obtained through Bader charge analysis.[79-81] In addition, the Jmol[82] program were used to build and visualize the systems and their vibrational frequencies, while VESTA[83] was employed to generate the images of the atomic structures.

Results:

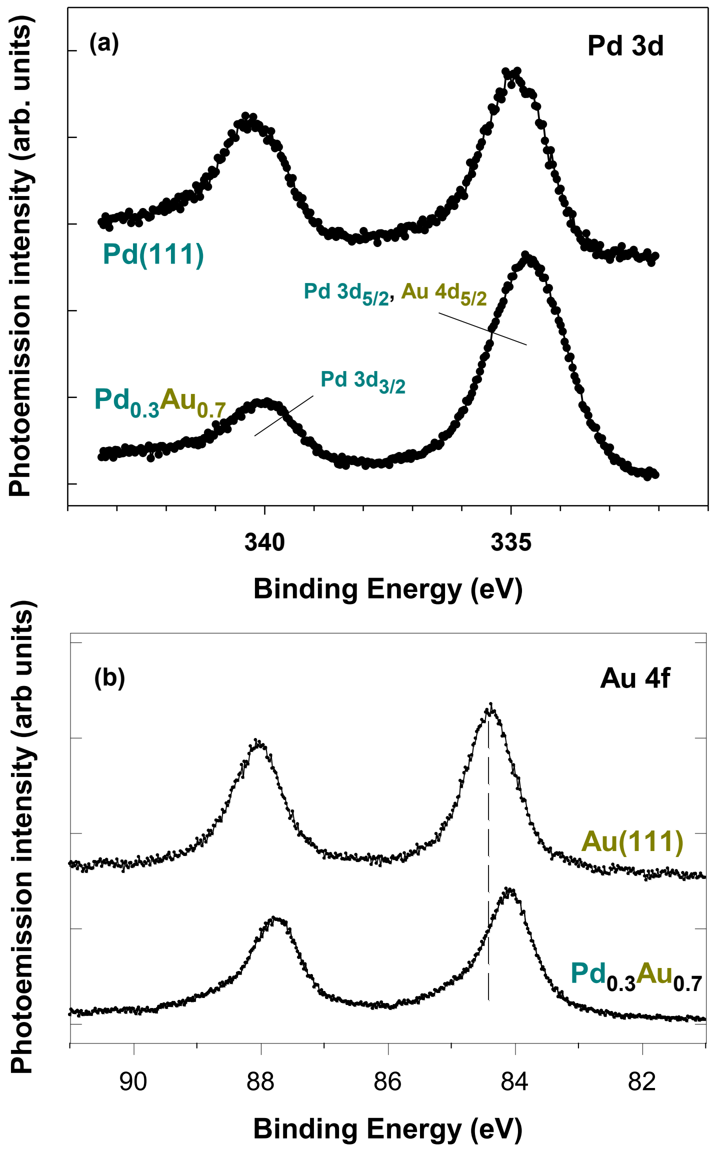

Figure S1. (a) Pd core-level spectra for Pd(111) and a $Pd_{0.3}Au_{0.7}$ alloy film. In the bimetallic system, the Pd $3d_{5/2}$ and Au $4d_{5/2}$ peaks exhibit overlap. (b) Au 4f core-level spectra for Au(111) and a $Pd_{0.3}Au_{0.7}$ alloy film. The alloy was generated by simultaneously deposition of 1.5 ML of Pd and 3.5 ML of Au onto a Mo(110) substrate, followed by annealing at 600–700 to promote mixing.

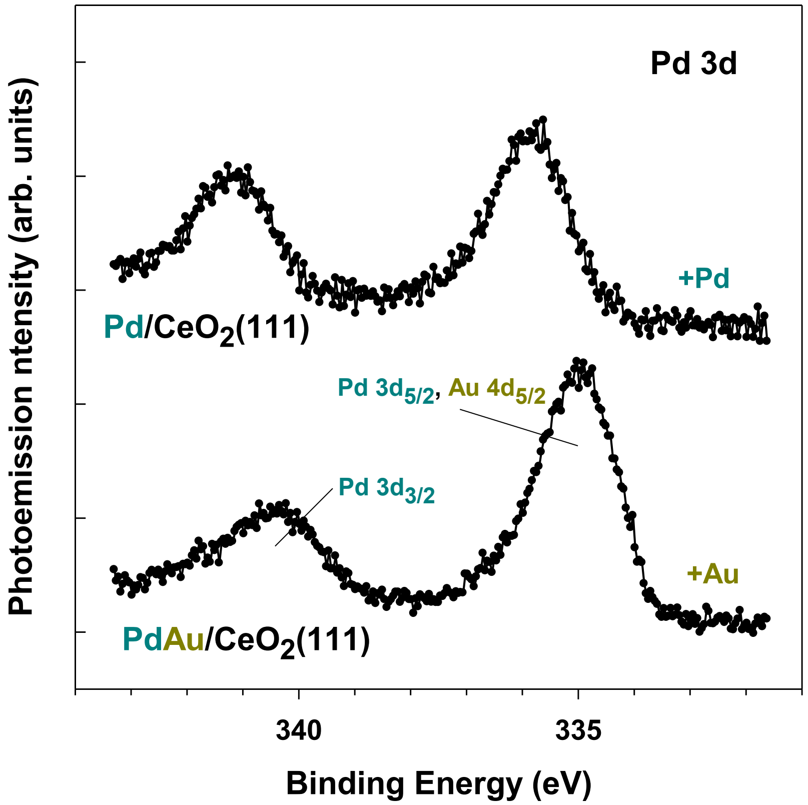

Figure S2. Core-level spectra for $Pd/CeO_2(111)$ and a $Pd_{0.3}Au_{0.7}/CeO_2(111)$ surface. In the bimetallic system, the Pd $3d_{5/2}$ and Au $4d_{5/2}$ peaks exhibit overlap. In the first step, 0.12 ML of palladium were vapor-deposited onto $CeO_2(111)$ at 300 K, followed by the deposition of 0.28 ML of Au and subsequent annealing at 600 K.

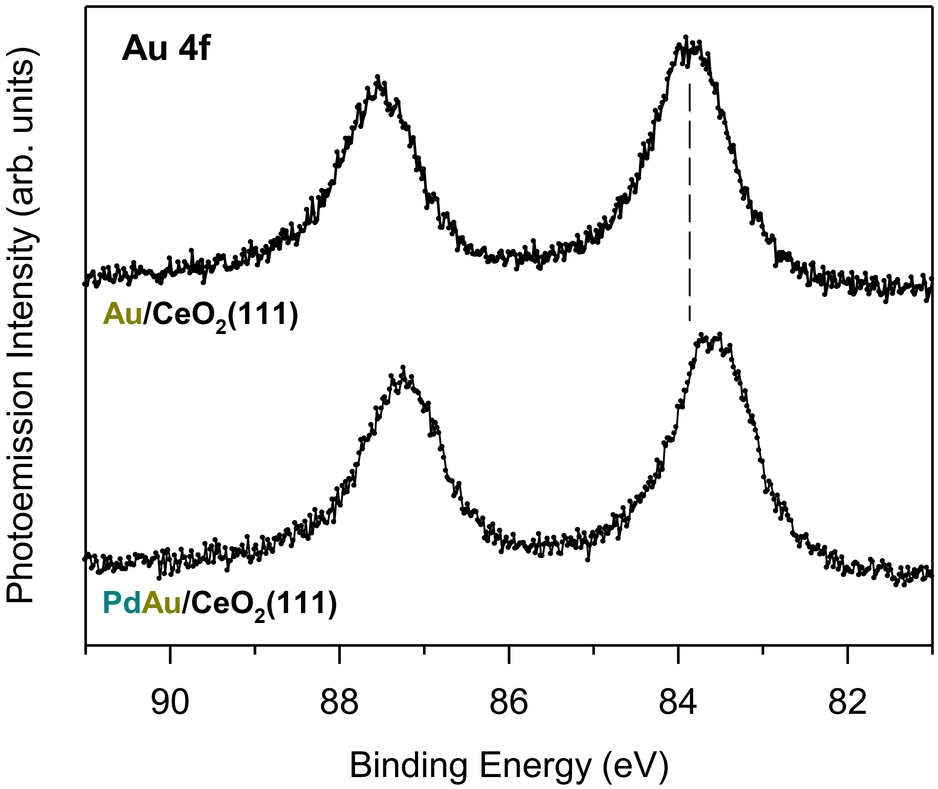

Figure S3. Au 4f core-level spectra for Au/CeO_2(111) and a $Pd_{0.3}Au_{0.7}$/CeO_2(111) surface. The content of gold in the Au/CeO_2(111) system is 0.28 ML. The Au 4f spectrum of $Pd_{0.3}Au_{0.7}$/CeO_2(111) was acquired in the same experiment that produced the Pd 3d and Au $4d_{5/2}$ spectra reported in Figure S2: In the first step, 0.12 ML of palladium were vapor-deposited onto CeO_2(111) at 300 K, followed by the deposition of 0.28 ML of Au and subsequent annealing at 600 K.

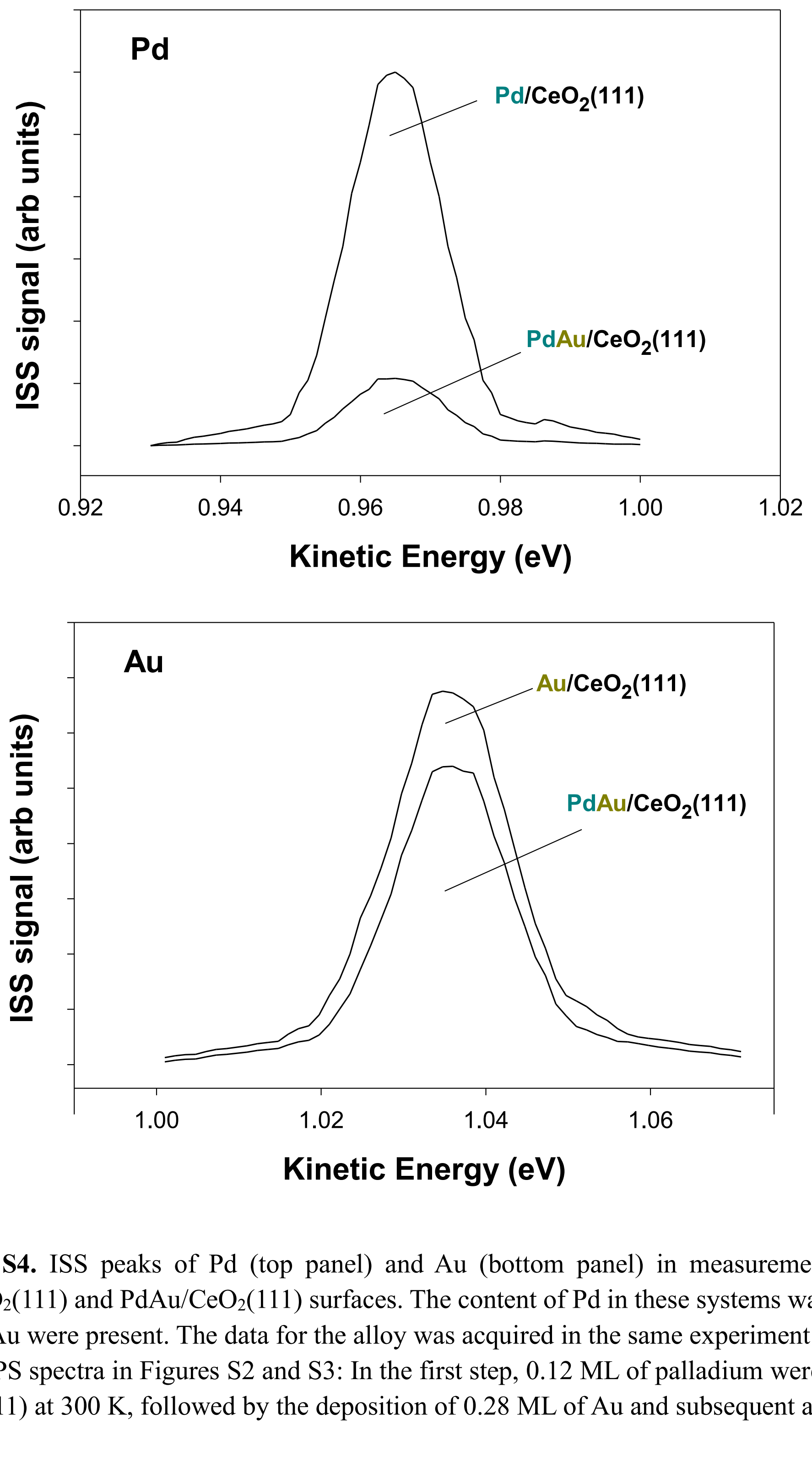

Figure S4. ISS peaks of Pd (top panel) and Au (bottom panel) in measurements for Pd/CeO_2(111), Au/CeO_2(111) and PdAu/CeO_2(111) surfaces. The content of Pd in these systems was 0.12 ML, while 0.28 ML of Au were present. The data for the alloy was acquired in the same experiment that produced the core level XPS spectra in Figures S2 and S3: In the first step, 0.12 ML of palladium were vapor-deposited onto CeO_2(111) at 300 K, followed by the deposition of 0.28 ML of Au and subsequent annealing at 600 K.

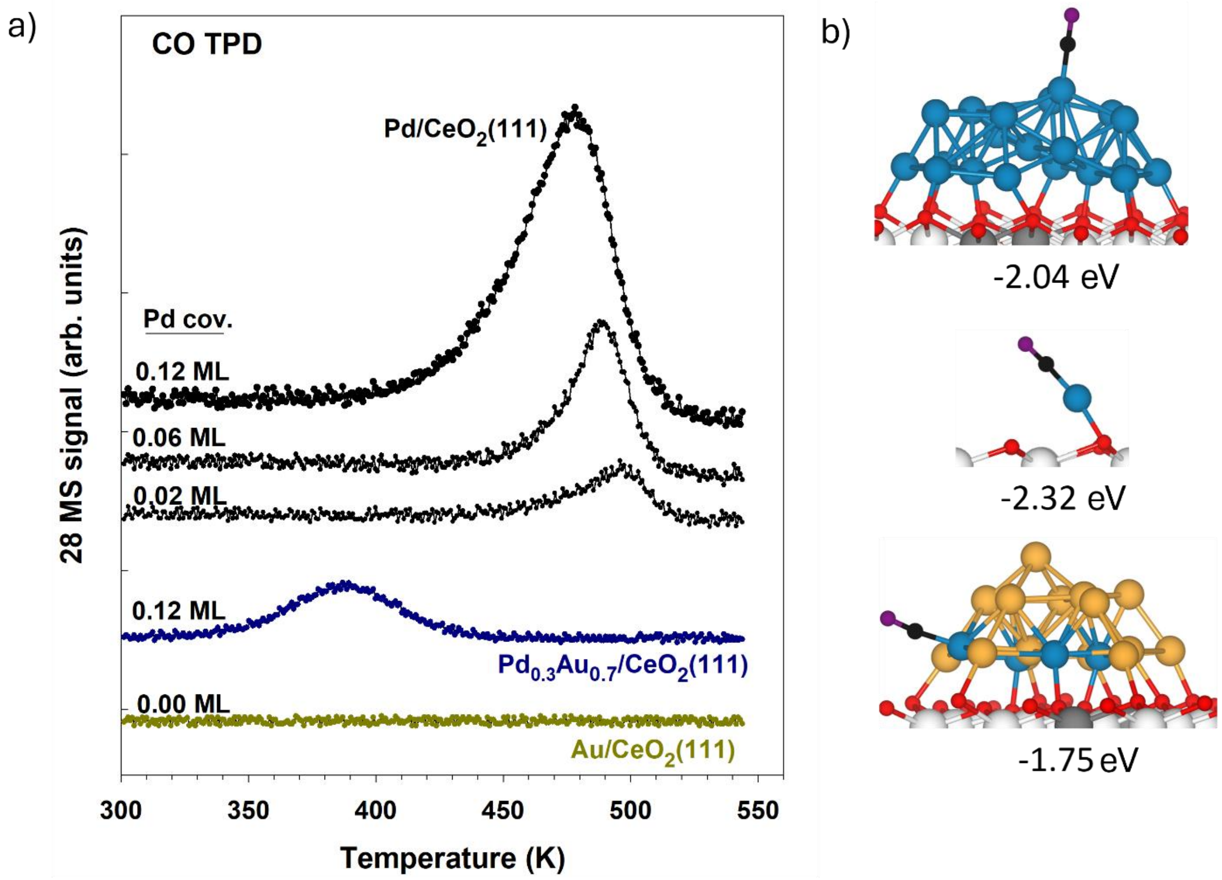

Figure S5. a) CO–TPD spectra collected after dosing 50 L of CO onto Pd/CeO_2(111), $Pd_{0.3}Au_{0.7}$/CeO_2(111), and Au/CeO_2(111) surfaces at room temperature. The heating rate was 5 K/s. The Pd coverage changed between 0.02 and 0.12 ML. The Pd-Au alloy was generated following the same methodology used for the experiments in Figure S2: 0.28 ML of Au were deposited on a CeO_2(111) surface pre-covered with 0.12 ML of Pd. The Au deposition was done at 300 K with subsequent annealing at 600 K. The Au/CeO_2(111) surface contained 0.40 ML of gold. b) DFT-calculated CO adsorption energies are shown alongside for representative models: Pd/CeO_2 (–2.32 eV), Pd_{20}/CeO_2 (–2.04 eV), and Pd_4Au_{16}/CeO_2 (–1.75 eV). Theoretical results reproduce the experimental trend in desorption temperature and intensity, confirming that Pd–Au alloying weakens CO binding due to electronic modification of the Pd sites.

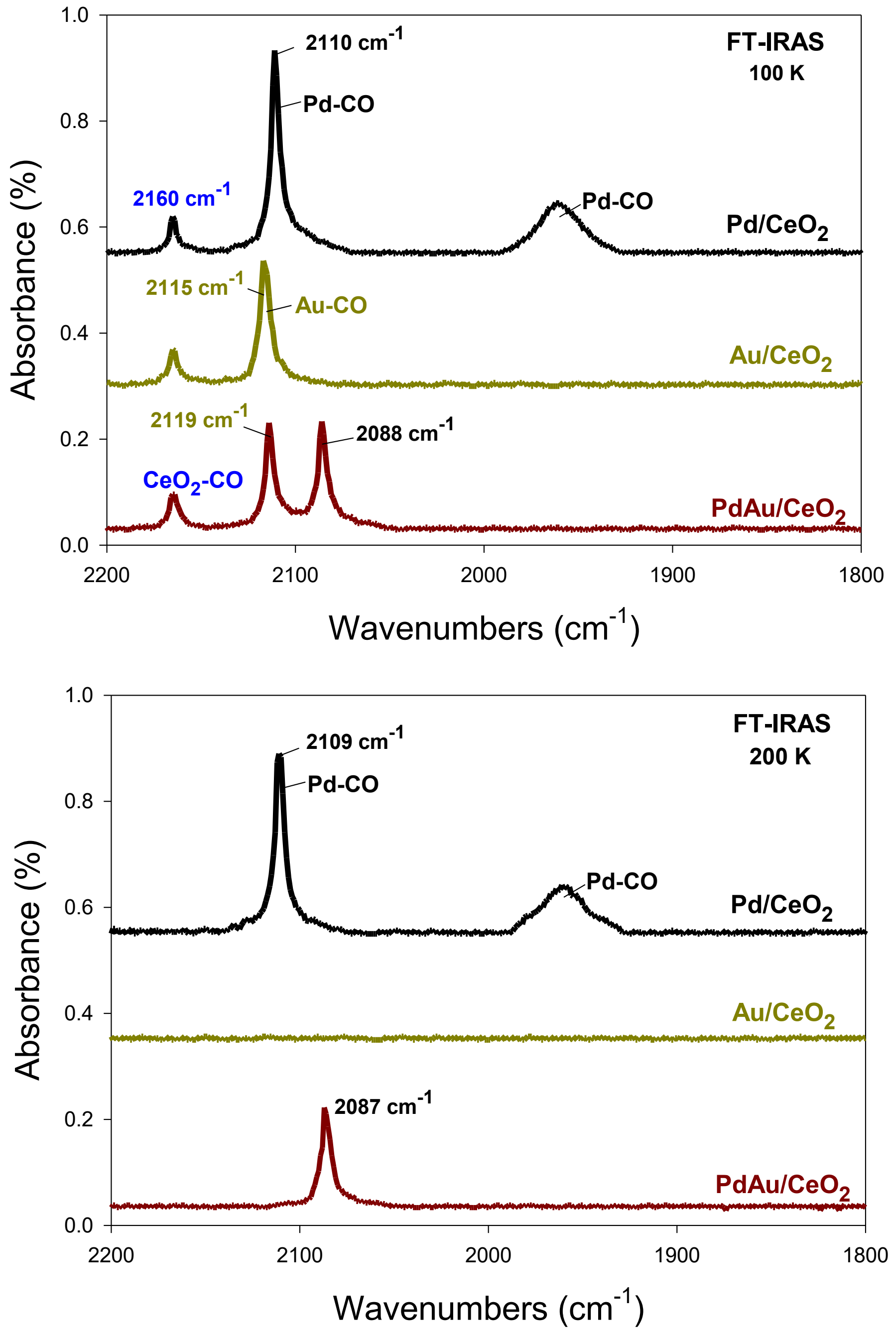

Figure S6. FT–IRAS spectra collected after dosing 50 L of CO to Pd/CeO_2(111), Au/CeO_2(111), $Pd_{0.3}Au_{0.7}$/CeO_2(111), and Au/CeO_2(111) surfaces at 100 K. For the top two spectra, the coverage of Pd or Au on the surface was 0.4 ML. The Pd-Au alloy was generated following the same methodology used for the experiments in Figure S2: 0.28 ML of Au were deposited on a CeO_2(111) surface pre-covered with 0.12 ML of Pd. The Au deposition was done at 300 K with subsequent annealing at 600 K. The bands associated with CO bonded to ceria or gold disappeared when the surfaces were heated to 200 K.

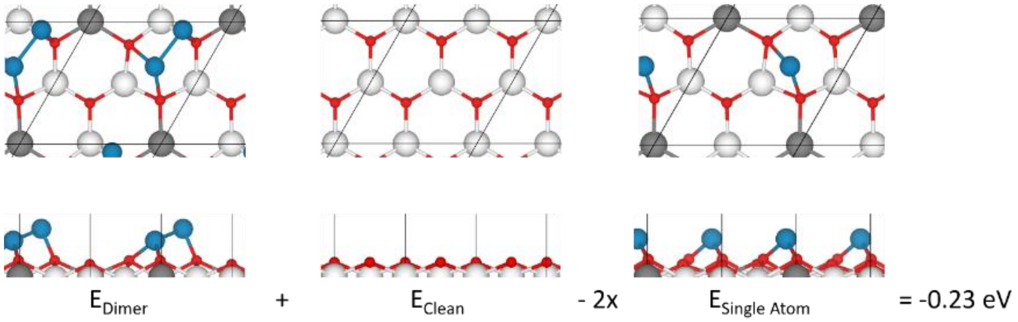

Figure S7: Relative stability of isolated Pd single atoms versus dimer configurations on the $CeO_2(111)$ surface. The total energy of two isolated Pd atoms adsorbed at low-coverage surface sites is taken as reference (0.00 eV). The formation of a Pd dimer is favored by –0.23 eV, indicating that aggregation into small clusters is thermodynamically preferred over single-atom dispersion. This energy difference was obtained by subtracting the total energy of the two isolated Pd atoms from that of the optimized dimer configuration. These results highlight the intrinsic instability of Pd single-atom catalysts on $CeO_2(111)$, which limits their practical relevance under reaction conditions.

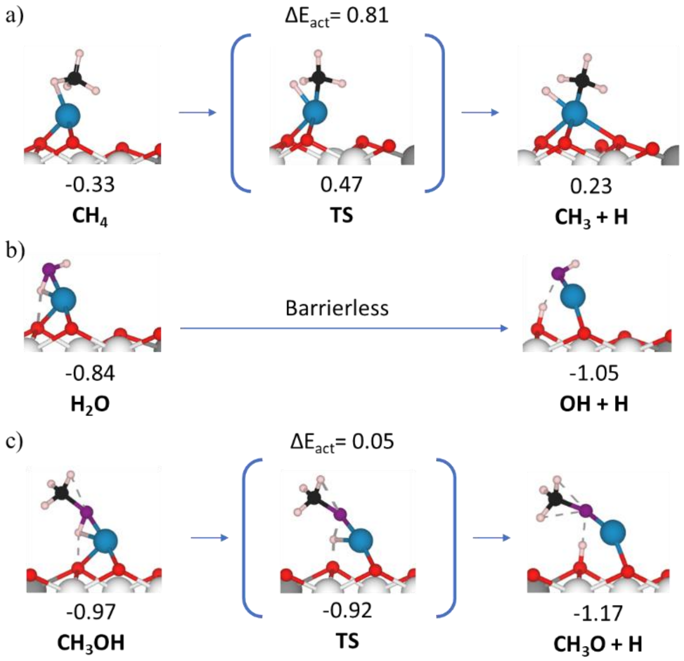

Figure S8: DFT-calculated reaction pathways for the activation of CH_4, H_2O, and CH_3OH on the $Pd_1/CeO_2(111)$ model. All energies are referenced to the $Pd_1/CeO_2(111)$ model with the corresponding gas-phase molecules. The results indicate that while single Pd atoms can activate all three molecules involved in the $CH_4 + H_2O \rightarrow CH_3OH$ process, they fail to inhibit further decomposition of methanol. This behavior is consistent with experimental observations showing excessive reactivity and poor selectivity on Pd/CeO_2 catalysts.

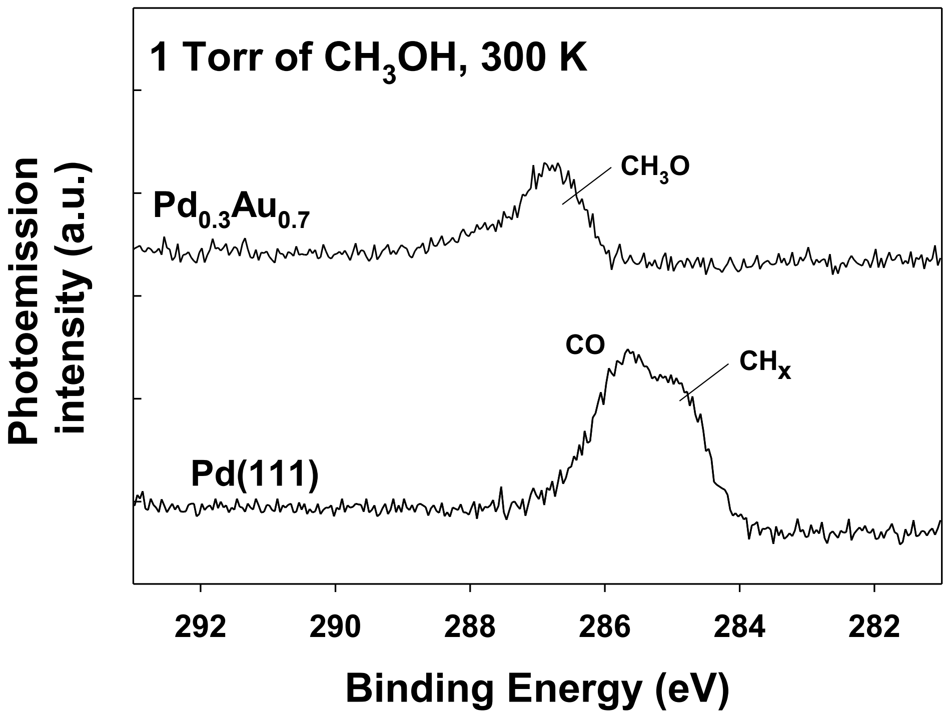

Figure S9. Dissociation of methanol on $Pd_{0.3}Au_{0.7}$ and Pd(111) surfaces.

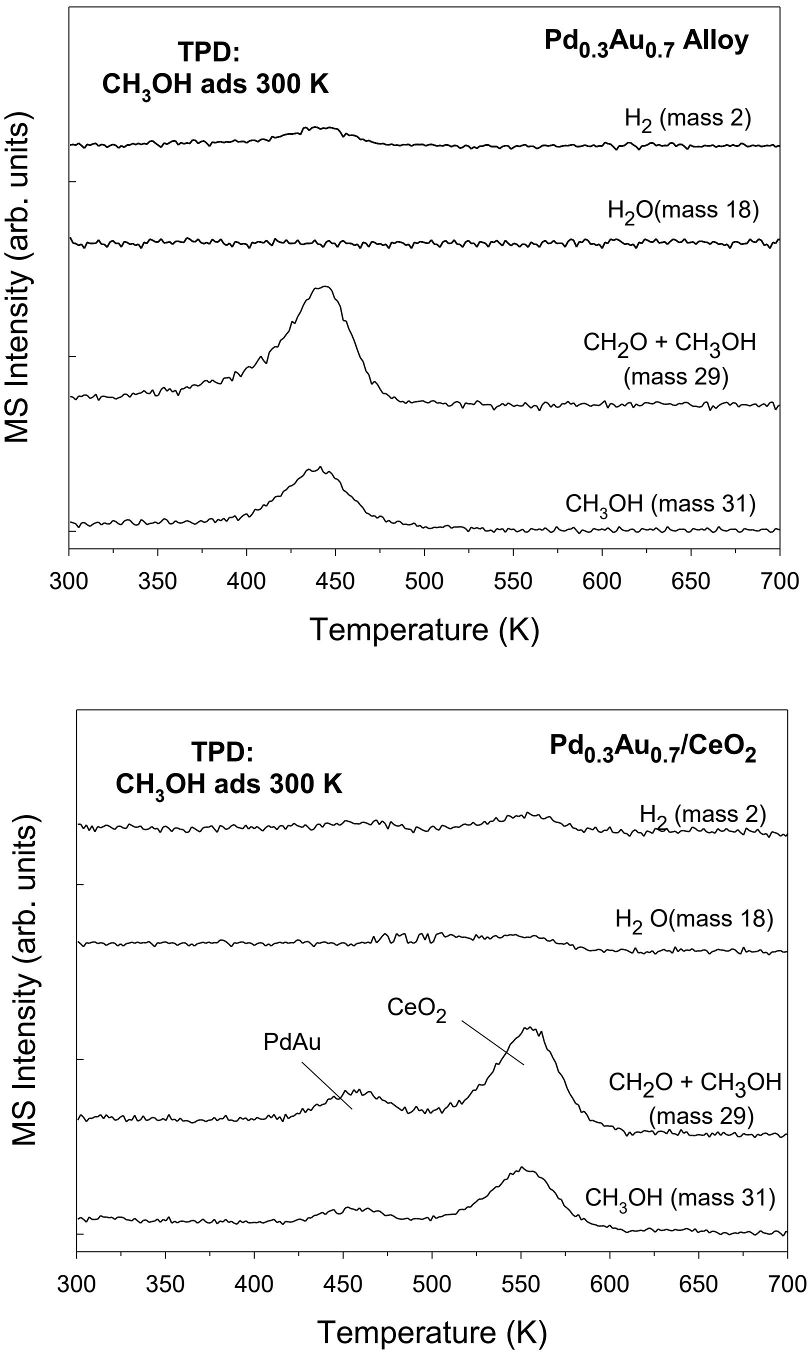

Figure S10. TPD spectra collected after adsorbing methanol on a $Pd_{0.3}Au_0$ alloy and on a $Pd_{0.3}Au_{0.7}/CeO_2(111)$ surface at 300 K. A heating rate of 5 K/s was used for these experiments. The Pd-Au alloy was generated following the same methodology used for the experiments in Figure S2: 0.28 ML of Au were deposited on a $CeO_2(111)$ surface pre-covered with 0.12 ML of Pd. The Au deposition was done at 300 K with subsequent annealing at 600 K.

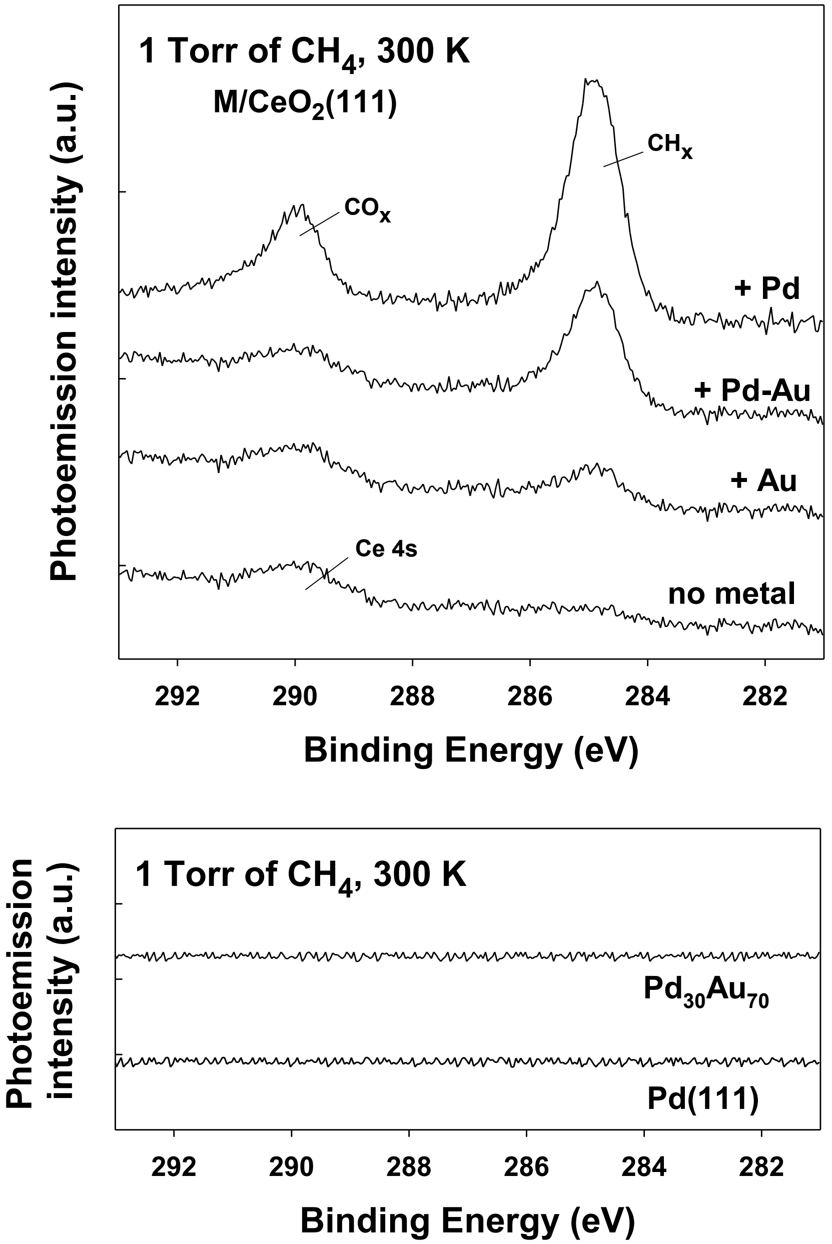

Figure S11. Dissociation of methane on different surfaces at room temperature. Pd(111), $Pd_{0.3}Au_{0.7}$(111) and CeO_2(111) do not facilitate methane dissociation. A minor degree of alkane dissociation is observed on Au/CeO_2(111). In contrast, Pd/CeO_2(111) and $Pd_{0.3}Au_{0.7}$/CeO_2(111) promote methane dissociation, leading to the formation of CH_x fragments. On Pd/CeO_2(111), full dissociation occurs ($CH_4 \rightarrow C + 4H$) along with the formation of CO_x. The Pd/CeO_2(111) and Au/CeO_2(111) systems contained 0.4 ML of the admetal. To generate the $Pd_{0.3}Au_{0.7}$/CeO_2(111) system, 0.28 ML of Au was deposited onto a CeO_2(111) surface pre-covered with 0.12 ML of Pd.

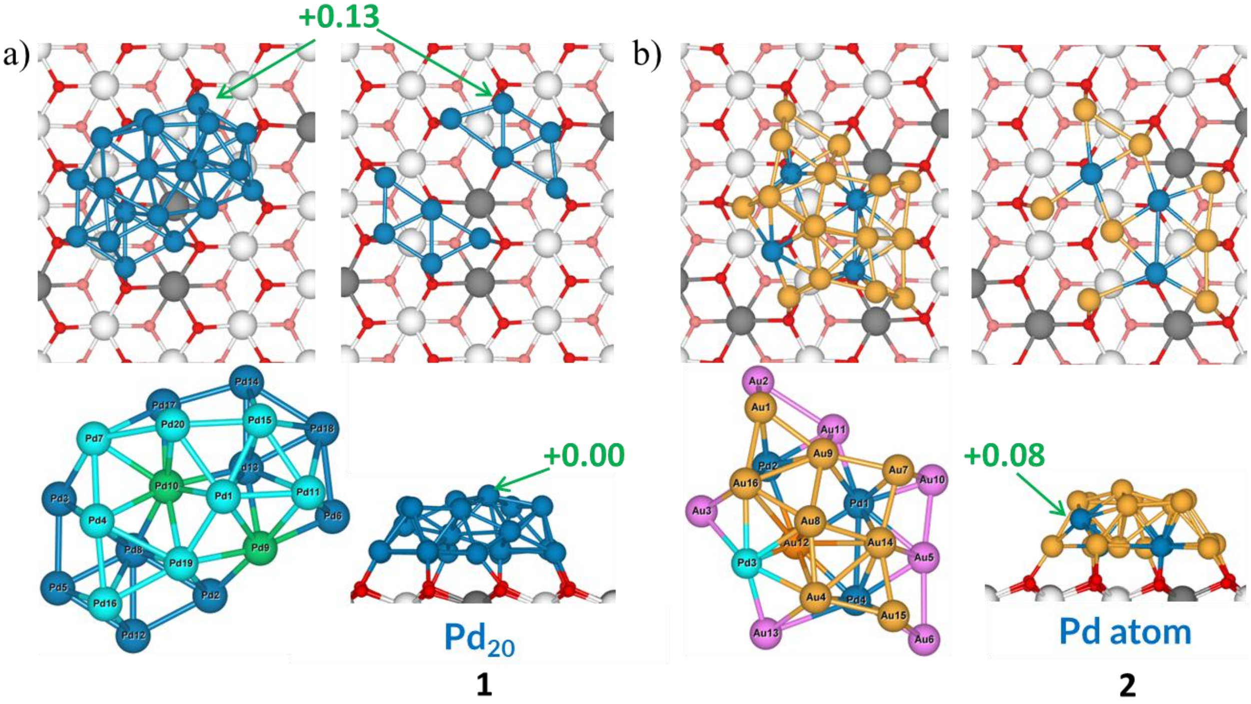

Figure S12. DFT-optimized final models used for (a) $Pd_{20}/CeO_2(111)$ and (b) the Pd atom model (see also text below). Top and side views are shown, along with a top view of the interfacial metal atoms only (upper-right corner). Bader charges of selected Pd sites are indicated (q $|e^{-}|$). Color scheme: Ce^{4+} (white), Ce^{3+} (gray), surface O (red), subsurface O (pink), Pd (blue), Au (gold). A zoomed-in top view of the cluster (bottom-left corner) includes atom labels and color coding for reference (see Table S1). Interfacial Au/Pd atoms are marked in pink/dark blue, second layer Au/Pd atoms in gold/light blue. Bottom-layer Au/Pd atoms highly coordinated but not interacting with surface oxygen atoms are shown in orange/green.

Table S1. Bader charges (q |e⁻|) for the Pd_4Au_{16} (left) and Pd_{20} (right) clusters from structures **1** and **2** in Figure S4. Interfacial Au/Pd atoms are marked in pink/dark blue, second layer Au/Pd atoms in gold/light blue. Bottom-layer Au/Pd atoms highly coordinated but not interacting with surface oxygen atoms are shown in orange/green.

**Atom**	**q(e⁻)**
Au1	-0.10
Au2	0.11
Au3	0.07
Au4	-0.06
Au5	0.12
Au6	0.09
Au7	-0.04
Au8	-0.01
Au9	-0.06
Au10	0.09
Au11	0.11
Au12	0.05
Au13	0.07
Au14	0.02
Au15	-0.05
Au16	-0.08
Pd1	0.29
Pd2	0.24
Pd3	0.08
Pd4	0.23
**∑q($Au^{\delta+}$)**	0.74
**∑q($Au^{\delta-}$)**	-0.40
**∑q(Au)**	0.34
**∑q(Pd)**	0.83
**q(Pd_4Au_{16})**	1.17

**Atom**	**q(e⁻)**
Pd1	0.00
Pd2	0.07
Pd3	0.09
Pd4	0.00
Pd5	0.13
Pd6	0.07
Pd7	-0.03
Pd8	0.20
Pd9	0.01
Pd10	0.04
Pd11	-0.05
Pd12	0.14
Pd13	0.28
Pd14	0.13
Pd15	-0.04
Pd16	-0.10
Pd17	0.12
Pd18	0.12
Pd19	-0.03
Pd20	-0.03
**∑q($Pd^{\delta+}$)**	1.41
**∑q($Pd^{\delta-}$)**	-0.29
**q(Pd_{20})**	1.12

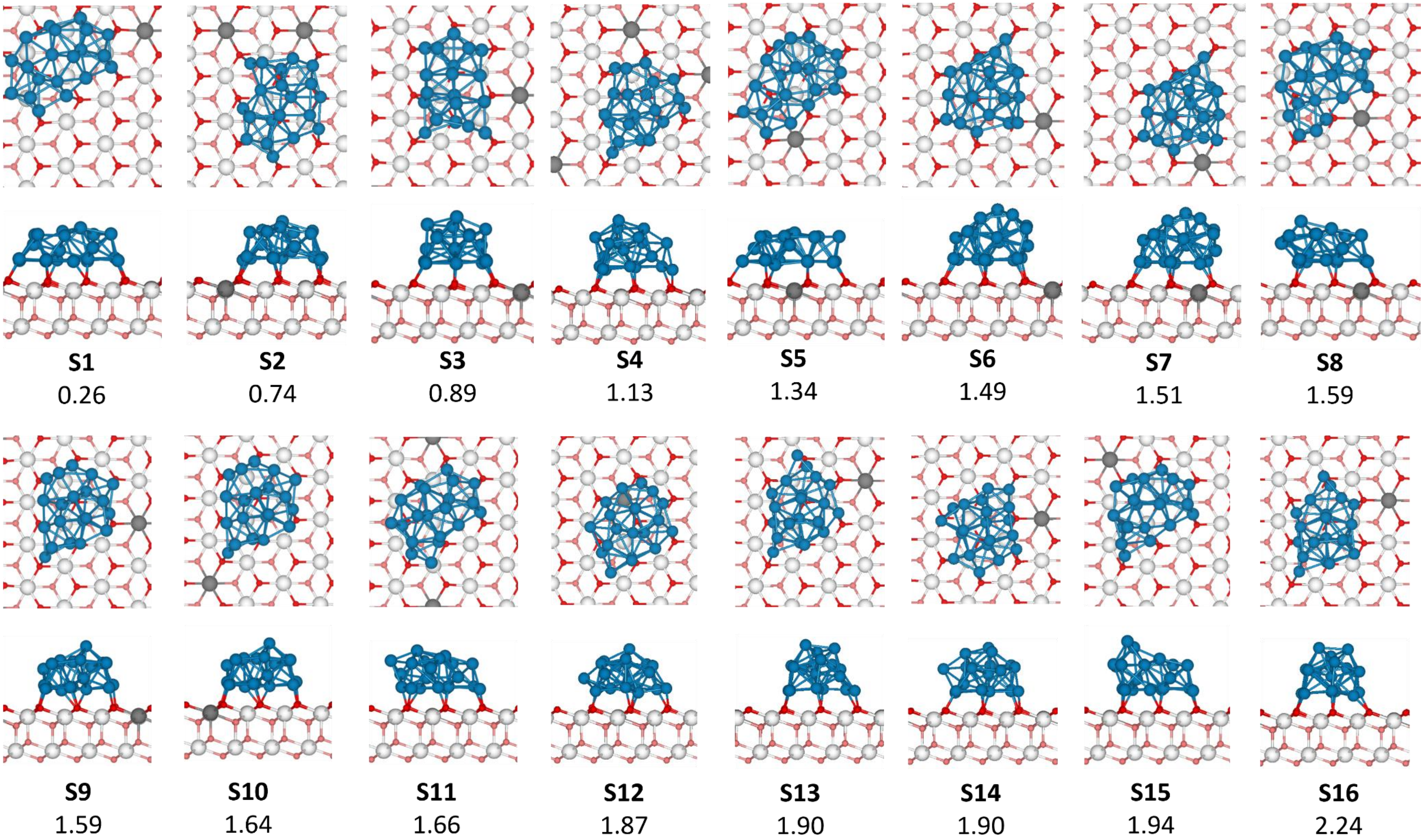

Figure S13. DFT-optimized structures for $Pd_{20}/CeO_2(111)$ after the GOFEE + VASP scheme. Relative energies are indicated in eV. Color scheme: Ce^{4+} (white), Ce^{3+} (gray), surface O (red), subsurface O (pink), Pd (blue).

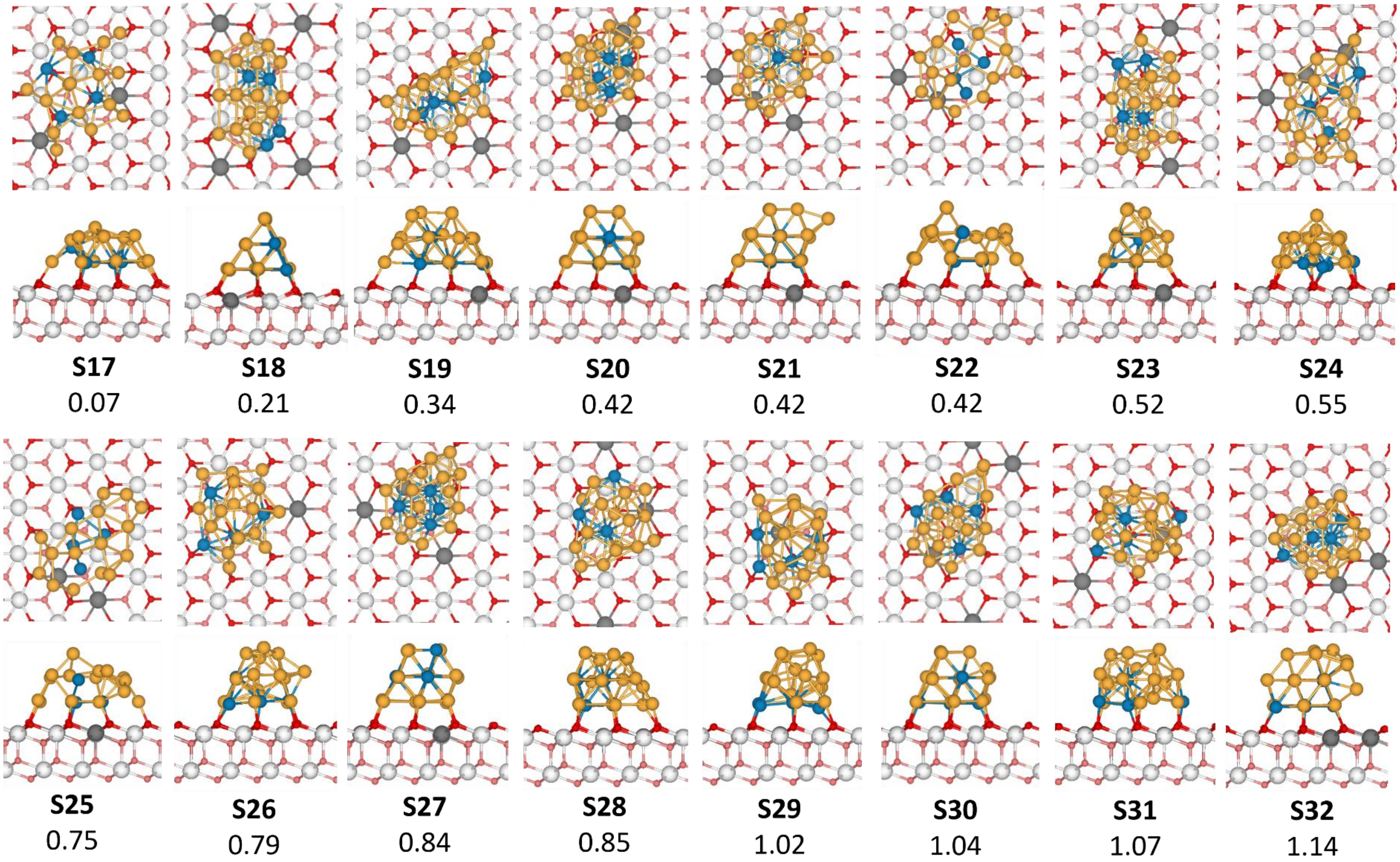

Figure S14. DFT-optimized structures for $Pd_4Au_{16}/CeO_2(111)$ after the GOFEE + VASP scheme. Relative energies are indicated in eV. Color scheme: Ce^{4+} (white), Ce^{3+} (gray), surface O (red), subsurface O (pink), Pd (blue), Au (gold).

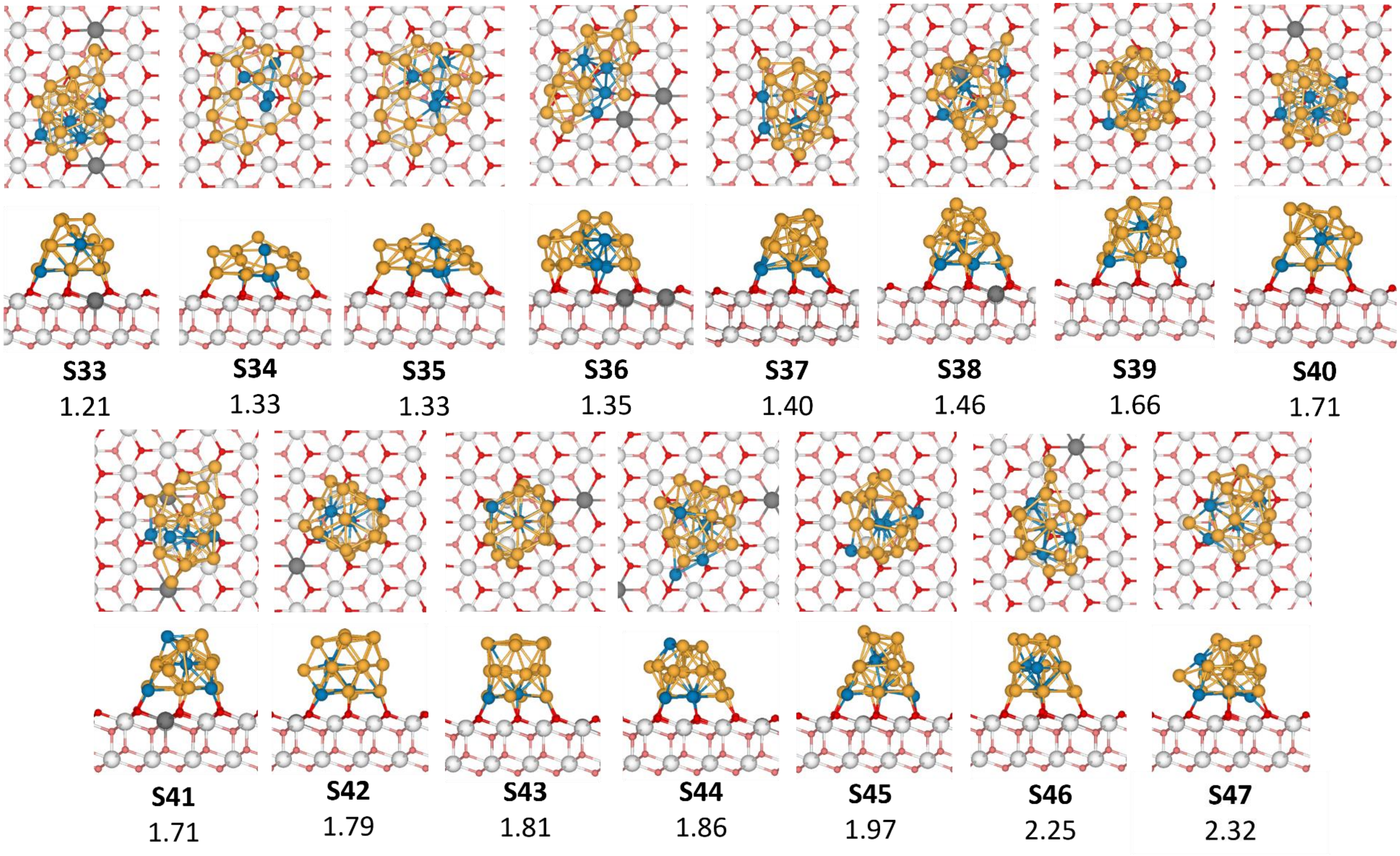

Figure S15. (Continues of Figure S6) DFT-optimized structures for $Pd_4Au_{16}/CeO_2(111)$ after the GOFEE + VASP scheme. Relative energies are indicated in eV.

Table S2. Relative energies (E_{rel}, in eV), Ce^{3+} positions, and cluster Bader charge (q(CL), in e^-) for the DFT-optimized $Pd_{20}/CeO_2(111)$ structures from Figures S4 and S5.

Structure	E_{rel}	Ce^{3+}	q(CL)
1	0.00	1, 2, 3	1.02
S1	0.26	1	0.50
S2	0.74	6, 16	0.80
S3	0.89	8	0.49
S4	1.13	1, 3	0.73
S5	1.34	14	0.49
S6	1.49	8	0.49
S7	1.51	4	0.35
S8	1.59	4	0.43
S9	1.59	8	0.49
S10	1.64	16	0.49
S11	1.66	3	0.46
S12	1.87	2	0.43
S13	1.90	1	0.45
S14	1.90	1	0.51
S15	1.94	15	0.46
S16	2.24	1	0.49

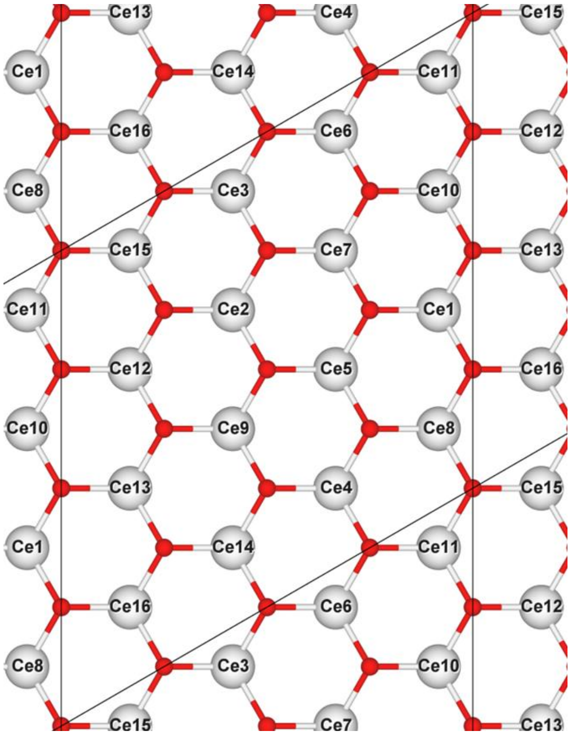

Scheme S1. Top view of the (4 × 4) slab of $CeO_2(111)$. Ce atoms labelled for reference.

Table S3. Relative energies (E_{rel}, in eV), Ce^{3+} positions, and cluster Bader charge (q(CL), in e^-) of the DFT-optimized $Pd_4Au_{16}/CeO_2(111)$ structures from Figures S4, S6 and S7.

Structure	E_{rel}	Ce^{3+}	q(CL)
2	0.00	5, 8, 11, 13	1.17
S17	0.07	5, 13	0.48
S18	0.21	4, 6, 13, 16	1.28
S19	0.34	4, 13	0.62
S20	0.42	4, 7	0.59
S21	0.42	4, 9, 12	0.87
S22	0.42	9, 12	0.57
S23	0.52	4, 6	0.62
S24	0.55	2, 7, 12	0.95
S25	0.75	4, 9	0.56
S26	0.79	1	0.26
S27	0.84	2, 4, 12	0.87
S28	0.85	3, 5	0.55
S29	1.02	2	0.31
S30	1.04	6, 9, 10	0.93
S31	1.07	5, 13	0.59
S32	1.14	4, 8	0.66
S33	1.21	4, 6	0.66
S34	1.33	0	-0.09
S35	1.33	0	-0.09
S36	1.35	4, 8	0.55
S37	1.40	0	0.02
S38	1.46	2, 4	0.62
S39	1.66	7	0.29
S40	1.71	3	0.32
S41	1.71	2, 14	0.56
S42	1.79	13	0.34
S43	1.81	1	0.35
S44	1.86	1	0.29
S45	1.97	2	0.32
S46	2.25	6	0.23
S47	2.32	0	-0.03

More than 2000 structures were explored at a lower level of theory using GOFEE, and the most stable ones were reoptimized with VASP at a higher level (see Modeling and DFT calculations). A lower number of close-energy isomers was obtained for Pd_{20}/CeO_2 than for Pd_4Au_{16}/CeO_2, and the latter tend to be taller. However, in both cases, bi- and tri-layered geometries composed from triangular (111)-like arrangements were identified, attempting to match the hexagonal symmetry of the oxygen atoms on the CeO_2(111) surface (Figures S7-S9). This indicates that in both systems, metal-oxygen interactions cause the first-layer metal atoms to follow the hexagonal symmetry of CeO_2(111), while atoms in upper layers stabilize through metal-metal bonds. For Pd_{20}, the most stable structure exhibits two flat Pd_5 trapezoidal patterns, where each Pd atom binds to the surface through an O atom. These two Pd_5 units remain separate, with the ten remaining Pd atoms forming a connecting bridge above them (Figure S6a). Similarly, the most stable structure found for Pd_4Au_{16}/CeO_2 (structure **2** in Figure S6b) adopts a bilayered, nearly symmetric star-shaped structure, where the interfacial metal atoms form at least one visible M_5 feature, though some metal-metal bonds are lost due to a combination of surface mismatch and the larger Ce^{3+}, which separate the metal atoms. This separation effect is also observed for Pd_{20}, explaining why the two Pd_5 units remain apart.

With a few high-energy exceptions (structures **S34**, **S35, S37** and **S47** in Figure S9), all clusters oxidize and produce a number of Ce^{3+} on the surface (Tables S2 and S3). The sum of Bader charges of the metal cluster correlates with the number of Ce^{3+} produced, with approximately 0.3–0.5e^- transferred for one Ce^{3+}, 0.5–0.6e^- for two, 0.7–0.9e^- for three, and more than 1e^- for four.

Interestingly, in the bimetallic structures, Pd atoms tend to locate at the interface between ceria and gold, where they become partially covered by Au, leaving one Pd atom accessible in

most cases. This is consistent with the experimental characterization of the $PdAu/CeO_2$ samples, which indicates that only ~20% of Pd are present at the surface. In fact, only four structures (**S23**, **S27**, **S29** and **S36**) exhibit accessible Pd dimers, and all are higher in energy (Figures S8-S9).

Finally, unless otherwise specified, the remaining structures studied and reported in subsequent sections preserve the Ce^{3+} concentration and distribution described in Figure S6, confirming the stability of these solutions and ensuring that the relative energies and reaction barriers reported remain consistent across all reaction pathways.

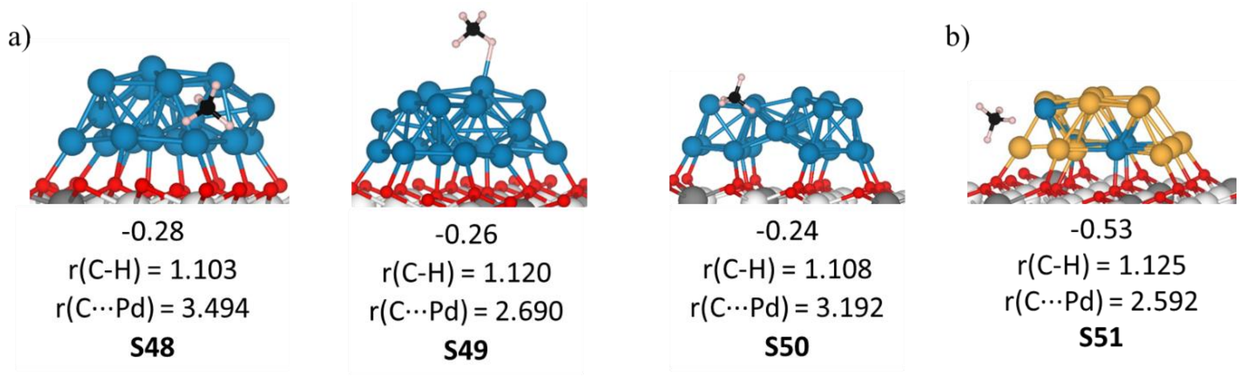

Figure S16. DFT-optimized structures for CH_4 adsorption on the (a) Pd_{20}, and (b) Pd atom models. Energies (in eV) are referenced to each model + CH_4 (gas), representing CH_4 adsorption energies. The distance (in Å) between C and the Pd adsorption site, as well as the longest C–H bond length, are also provided. Color scheme: Ce^{4+} (white), Ce^{3+} (gray), surface O (red), subsurface O (pink), Pd (blue), Au (gold), H (small white atoms), C (black). Calculated r(C–H) for gas phase methane is 1.095 Å.

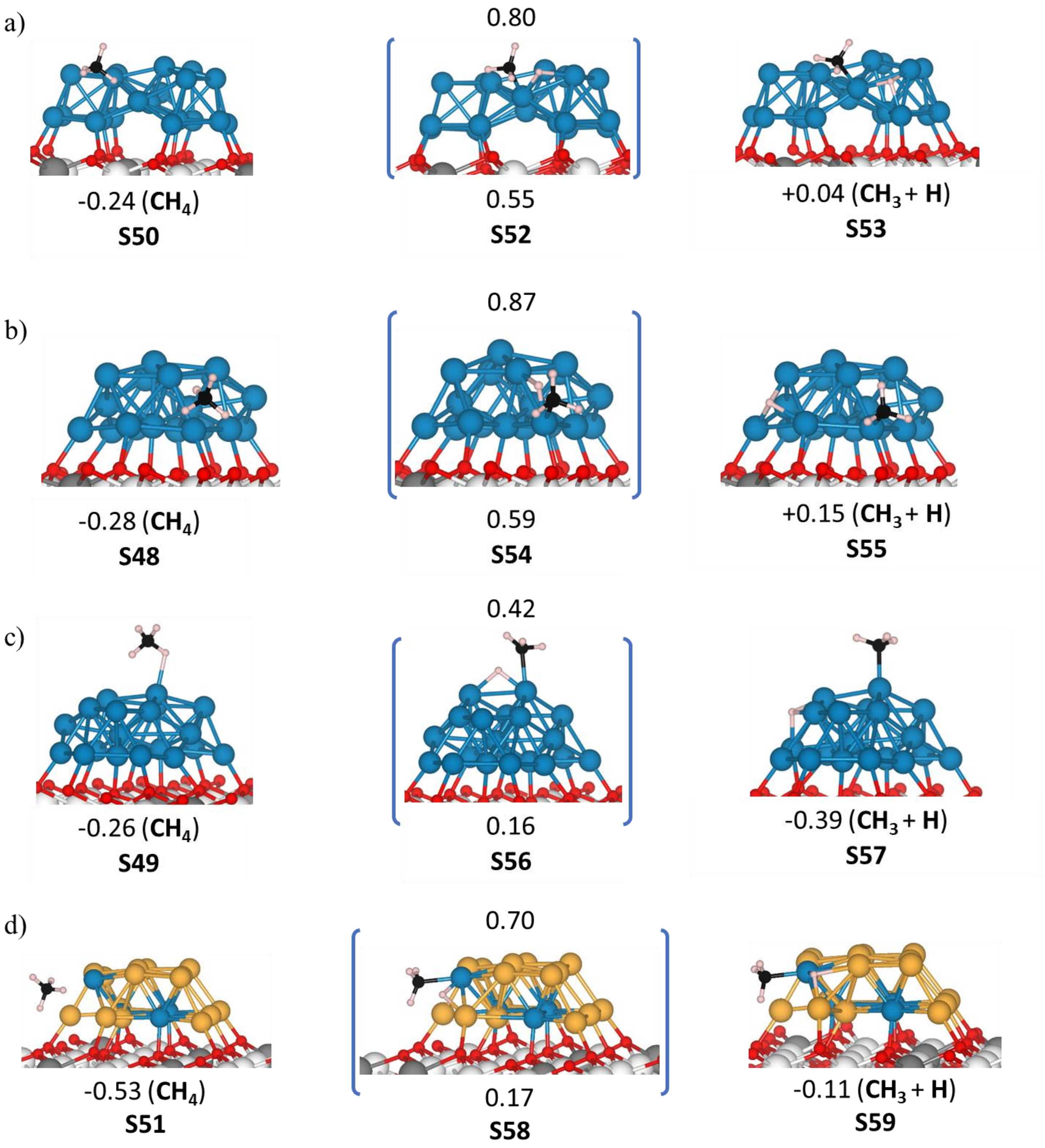

Figure S17. DFT-optimized structures for CH_4 adsorption and dissociation on (a-c) different Pd atoms of the Pd_{20} model and (d) on the Pd atom of the Pd_4Au_{16} model. Energies (in eV) referred to each model + CH_4 (gas) are indicated. Activation energies are indicated above the transition state structures. Color scheme: Ce^{4+} (white), Ce^{3+} (gray), surface O (red), subsurface O (pink), Pd (blue), Au (gold), H (small white atoms), C (black).

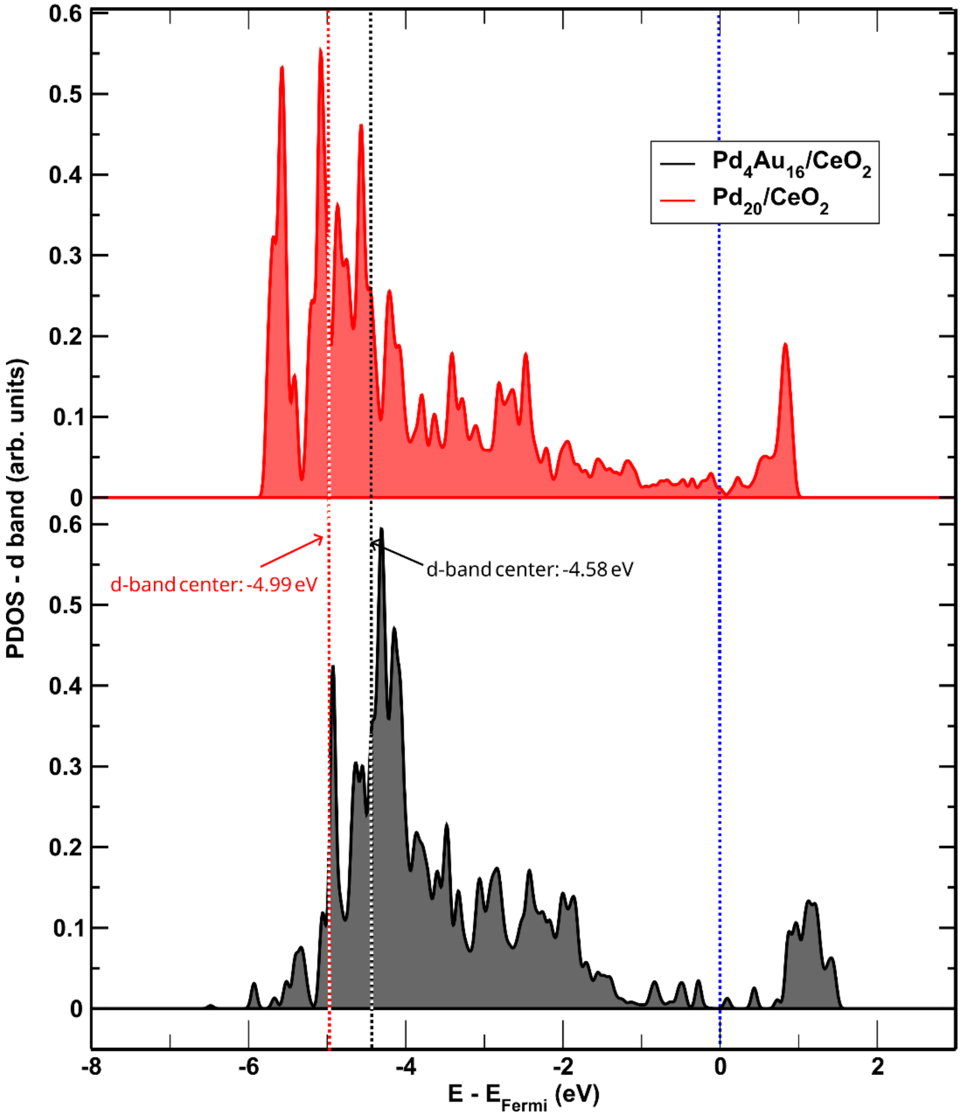

Figure S18. Atom- and *d*-orbital-projected density of states (PDOS) for the active Pd site in the Pd_4Au_{16} (black) and Pd_{20} (red) models (Pd3 and Pd10 in structures **1** and **2** in Figure S12, respectively).

CH_4 pre-activation in the Pd atom model is accompanied by an activation barrier that is 0.28 eV higher than in Pd_{20} (Figures 2 and S11c). This difference is primarily attributed to the stability of the dissociated state (CH_3 + H), as reflected in the reaction energy: in the Pd atom model, the reaction is endothermic by 0.42 eV, whereas in Pd_{20}, it is exothermic by –0.13 eV. This

behavior is largely influenced by the presence of Au. To clarify this effect, we separately calculated the adsorption energies of CH_3 and H species (Figure S13). The results show that H adsorption at a bridge site between two Au atoms is 0.45 eV less stable than at an Au-Pd bridge site in Pd_4Au_{16}, and 0.27 eV less stable than at a Pd-Pd site in Pd_{20}. Similarly, CH_3 adsorption is 0.15 eV more stable on a Pd atom than on an Au atom in the Pd atom model. These findings suggest that CH_3 and H species compete for the same active site in the Pd_4Au_{16} cluster model. Since only one exposed Pd atom is available in the Pd atom model, its overall activation capability is limited. Notably, in the Pd atom model, both CH_3 and H adsorb onto the same Pd atom, whereas in Pd_{20}, they occupy separate Pd atoms (Figures 2 and S11). Finally, the co-adsorption of CH_3 and H on the Pd atom model resulted in an energy of –0.11 eV, making it 0.74 eV more stable than the co-adsorption on Au atoms in the model (Figure S13f).

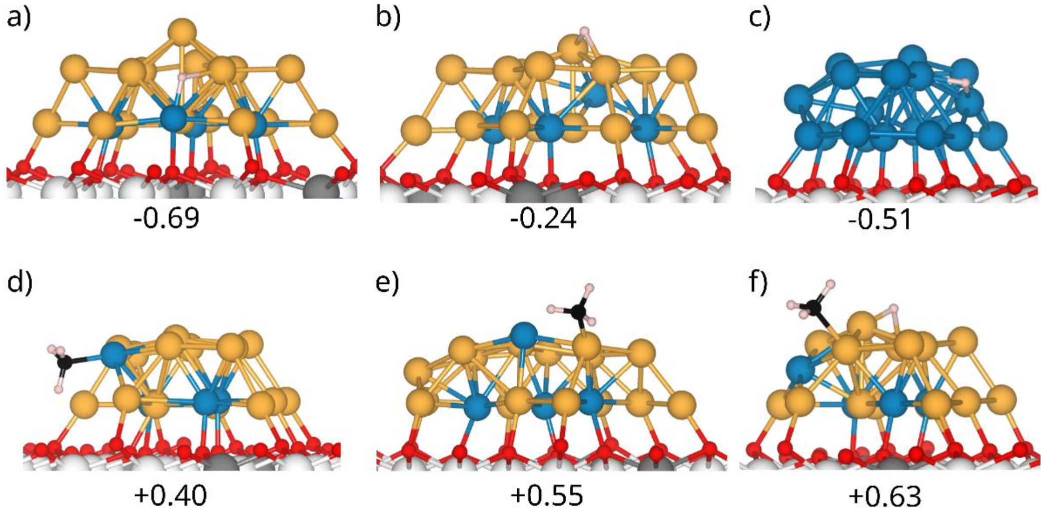

Figure S19. DFT-optimized structures of H adsorbed on (a) a bridge position between an Au and a Pd atom, and b) on a bridge position between two Au atoms in the Pd atom. c) H adsorbed on a bridge position between two Pd atoms in the Pd_{20} cluster. The adsorption energies for cases (a–c) are referenced to pristine models with ½ H_2 (gas). Adsorption of CH_3 on a (d) Pd atom, and (e) Au atom in the Pd atom model. The energies in (d–e) are calculated relative to the reaction CH_4 (gas) → CH_3* + ½ H_2 (gas). (f) Co-adsorption of CH_3* and H* on different Au atoms, with H adsorbed in a bridge position. The energy in (f) is referenced to the pristine Pd atom model with CH_4 (gas).

All energies in eV. Color scheme: Ce^{4+} (white), Ce^{3+} (gray), surface O (red), subsurface O (pink), Pd (blue), Au (gold), H (small white atoms), C (black).

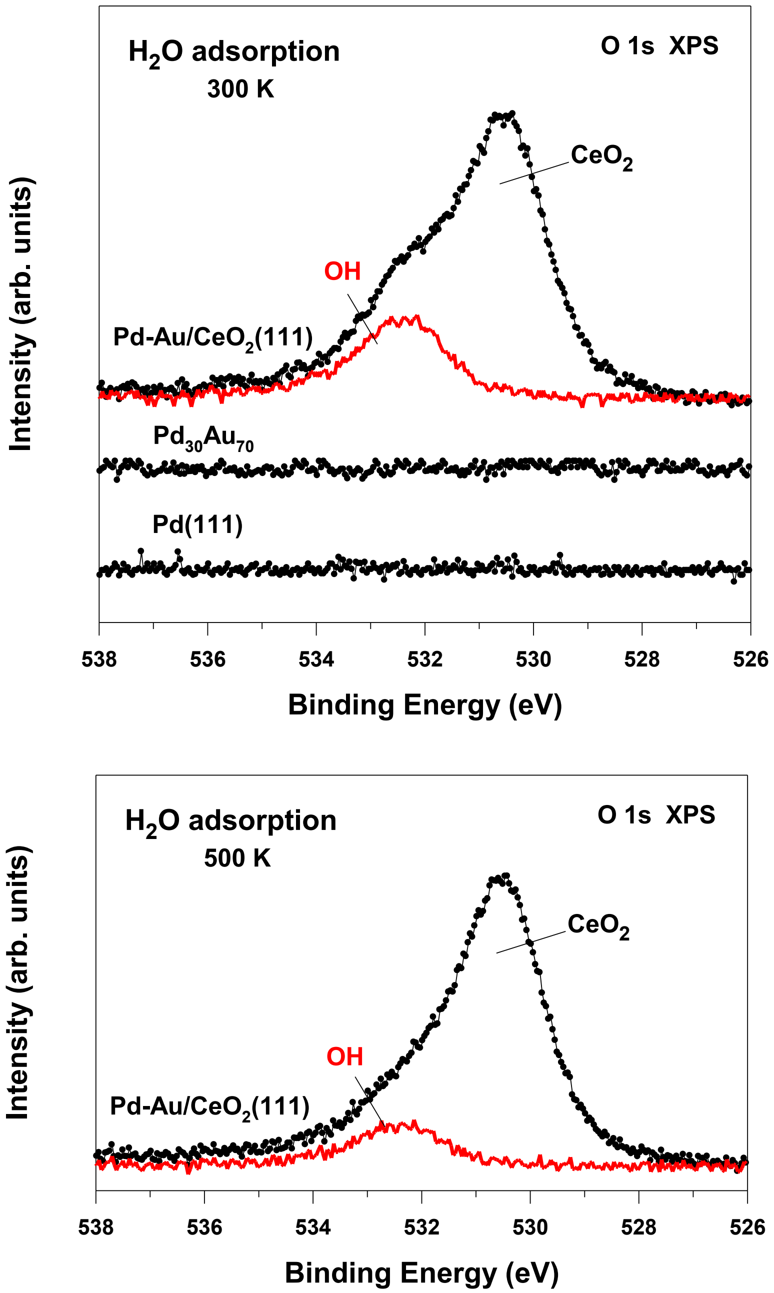

Figure S20. Reaction of water with different surfaces at room temperature and 500 K. All surfaces were exposed to 1 Torr of H_2O. Pd(111) and $Pd_{0.3}Au_{0.7}$(111) do not adsorb or dissociate water molecules. The red trace represents the difference spectrum obtained by susbtracting the O 1s spectra of $Pd_{0.3}Au_{0.7}/CeO_2$(111) from that of $H_2O/Pd_{0.3}Au_{0.7}/CeO_2$(111). To generate the $Pd_{0.3}Au_{0.7}/CeO_2$(111) system, 0.28 ML of Au was deposited onto a CeO_2(111) surface pre-covered with 0.12 ML of Pd.

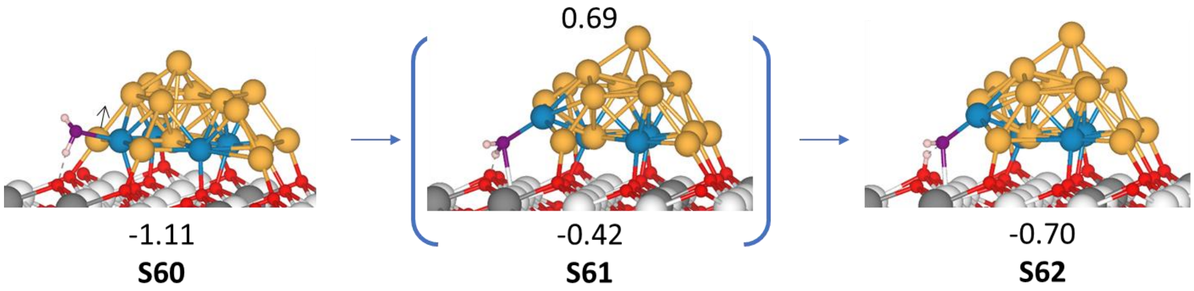

Figure S21. DFT-optimized structures for H_2O adsorption and dissociation on the Pd atom model. Energies (in eV) are referenced to the Pd atom model + CH_4(gas) + H_2O(gas). Activation energies are indicated above the transition state structure. The black arrow in structure **S60** highlights the movement of an Au atom, creating space for dissociation. Color scheme: Ce^{4+} (white), Ce^{3+} (gray), O (red), Pd (blue), Au (gold), O from H_2O (purple), H (small white atoms).

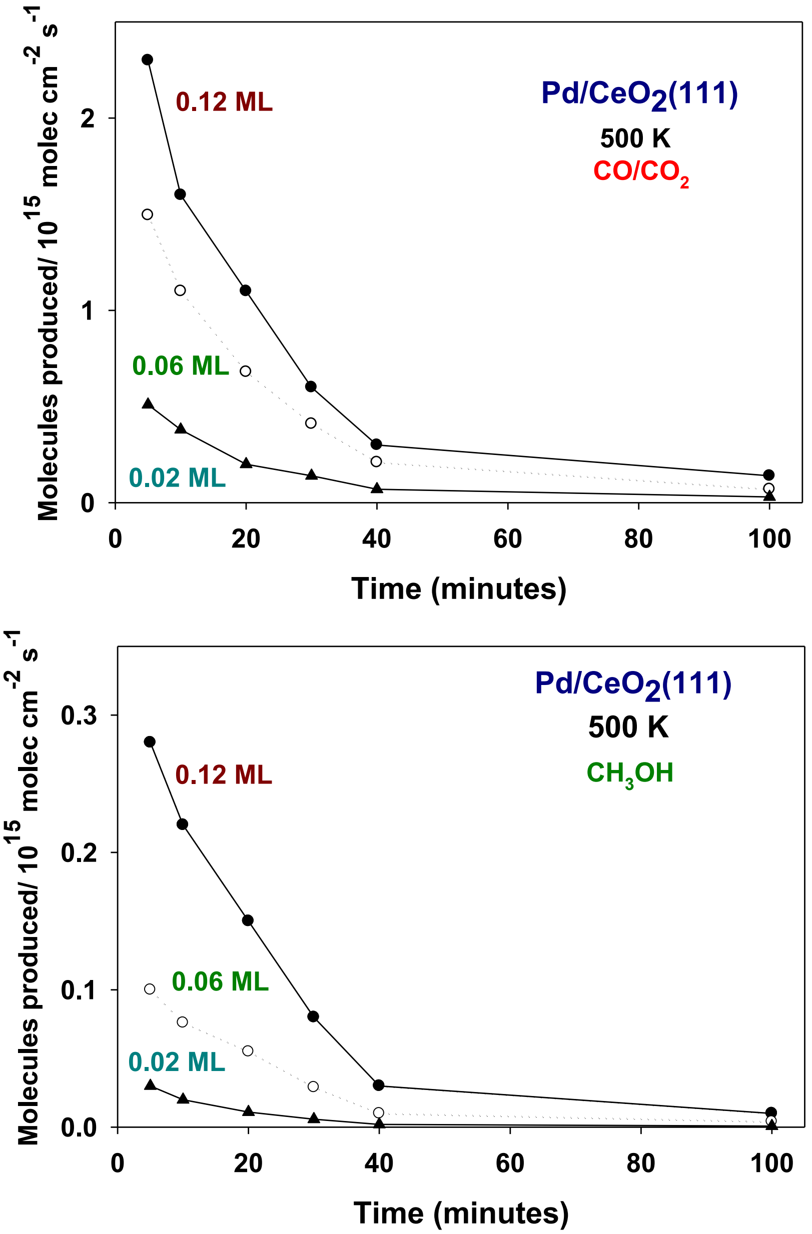

Figure S22. Conversion of methane (1 Torr) by reaction with water (1 Torr) at 500 K over a series of Pd/CeO_2(111) catalysts with admetal coverages of 0.02, 0.06 and 0.12 ML. The top panel shows the production of CO/CO_2 (dominant products), while the production of CH_3OH (minority product) is shown in the bottom panel.

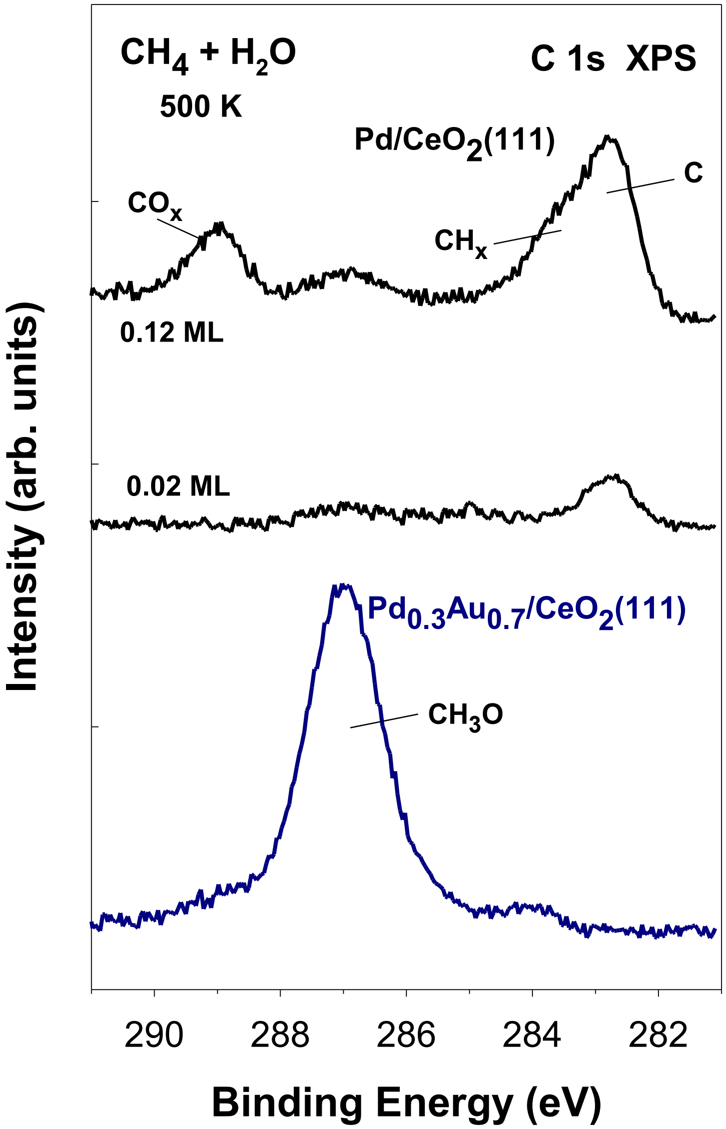

Figure S23. C 1s XPS spectra collected after performing the reaction of methane with water on Pd/CeO_2(111) and $Pd_{0.3}Au_{0.7}$/CeO_2(111) catalysts. Coverages of 0.12 and 0.02 ML were deposited on the ceria substrate. The surfaces were exposed to methane (1 Torr) and water (1 Torr) at 500 K for 40 minutes before pumping out these gases and collecting the C 1s spectra. The Pd-Au alloy was generated following the same methodology used for the experiments in Figure S2: 0.28 ML of Au were deposited on a CeO_2(111) surface pre-covered with 0.12 ML of Pd. The Au deposition was done at 300 K with subsequent annealing at 600 K before studying the CH_4 + H_2O reaction.

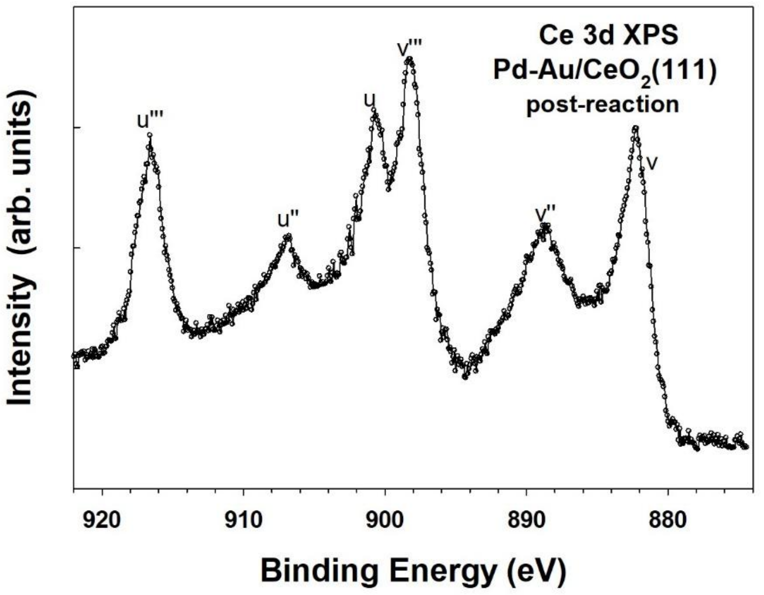

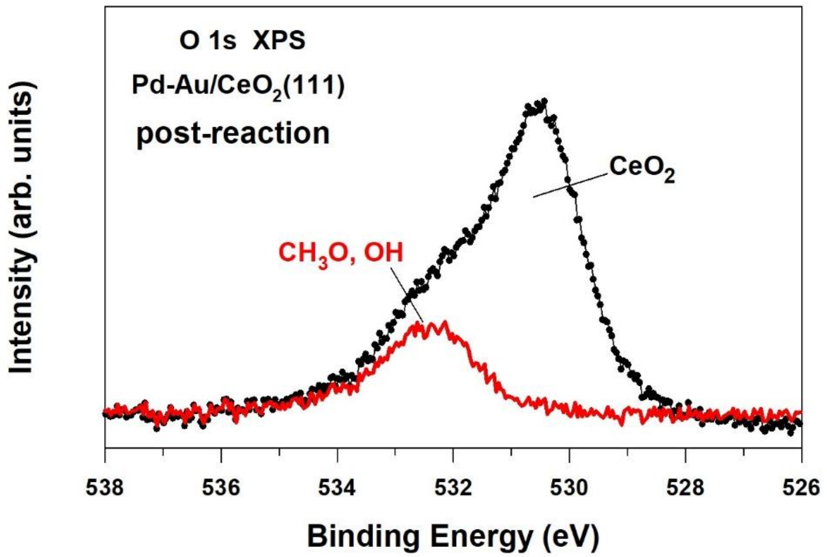

Figure S24. Ce 3d and O 1s spectra collected after performing the reaction of methane with water on a $Pd_{0.3}Au_{0.7}/CeO_2(111)$ catalysts. The surface was exposed to methane (1 Torr) and water (1 Torr) at 500 K for 40 minutes before pumping out these gases and collecting the Ce 3d and O 1s spectra. The Pd-Au alloy was generated following the same methodology used for the experiments in Figure S2: 0.28 ML of Au were deposited on a $CeO_2(111)$ surface pre-covered with 0.12 ML of Pd. The Au deposition was done at 300 K with subsequent annealing at 600 K before studying the $CH_4 + H_2O$ reaction.

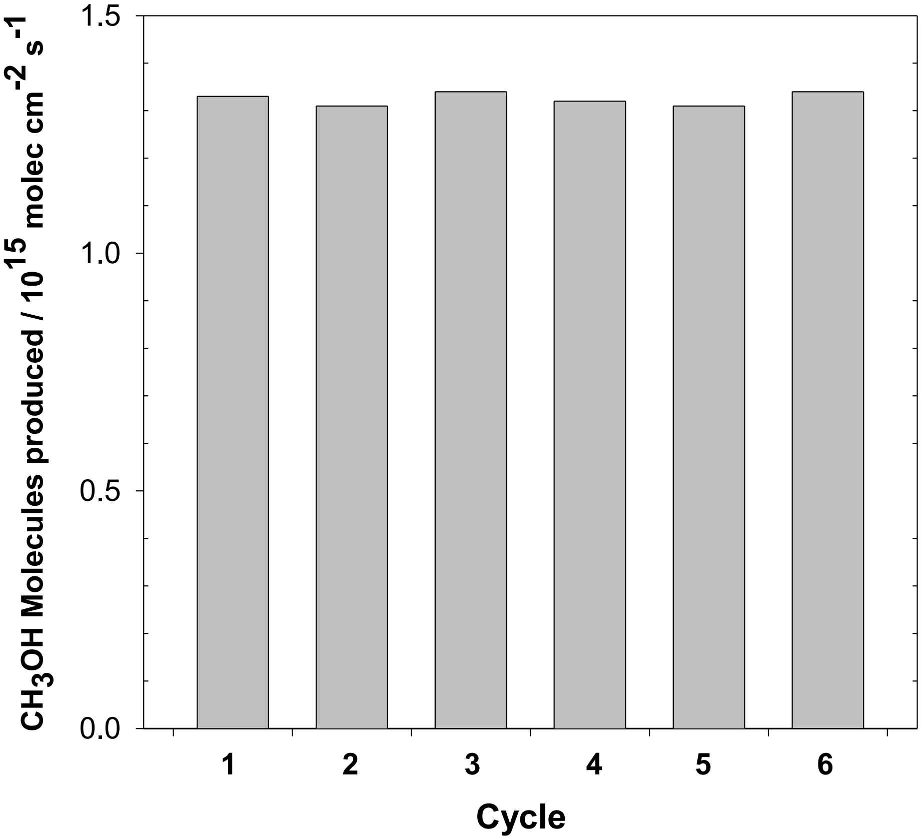

Fig S25 Conversion of methane (1 Torr) by reaction with water (1 Torr) at 500 K over $Pd_{0.3}Au_{0.7}/CeO_2(111)$ catalyst after several cycles of operation. Each cycle of operation lasted 30 minutes. The alloy was generated following the same methodology used for the experiments in Figures S2 and S3: 0.28 ML of Au were deposited on the $Pd/CeO_2(111)$ surface at 300 K with subsequent annealing at 600 K.

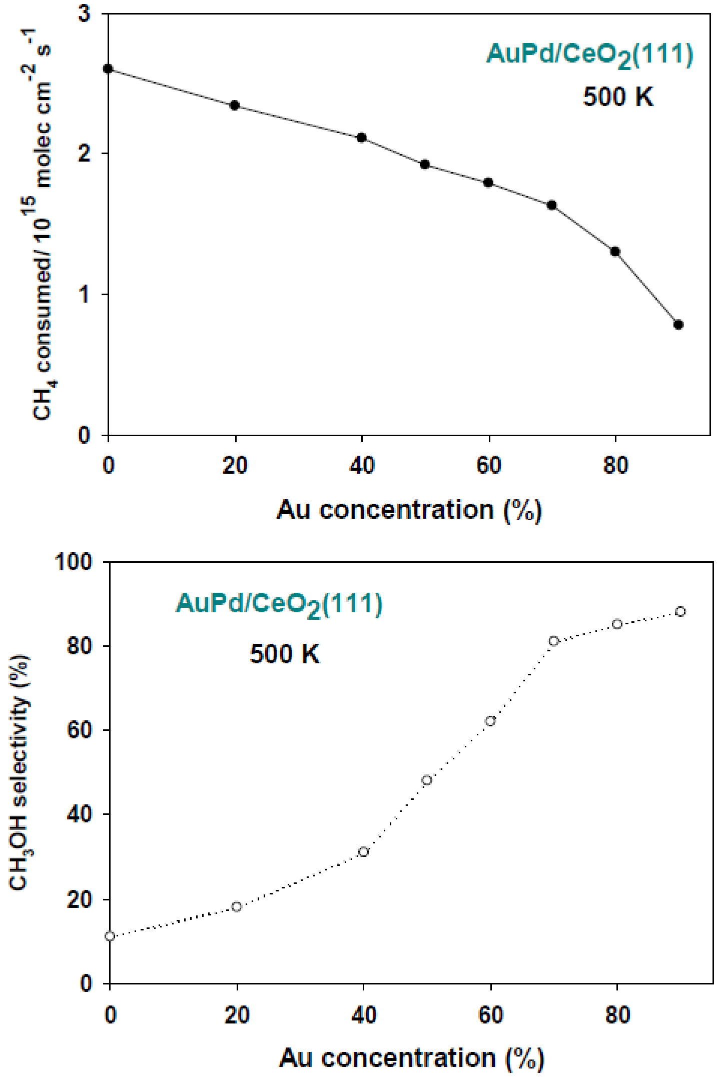

Figure S26. Effect of Au concentration on methane conversion (top) and methanol selectivity (bottom). Values were obtained after 5 minutes of reaction with 1 Torr of methane and 1 Torr of water at 500 K.

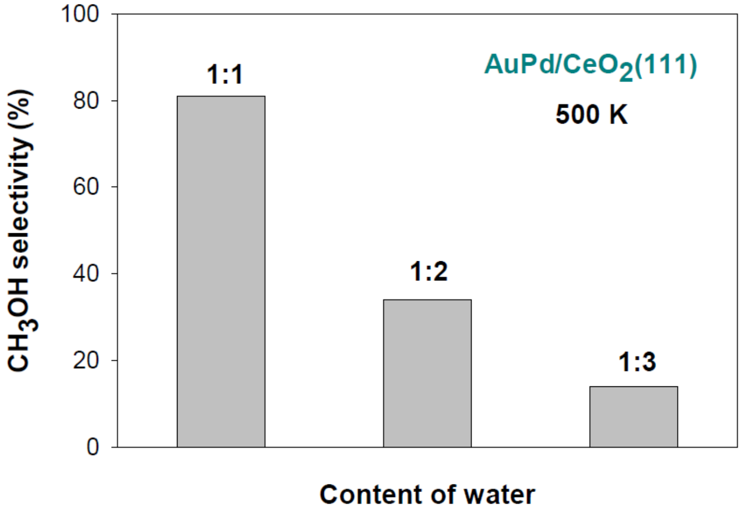

Figure S27. Effect of water content on methanol selectivity for the $Pd_{0.3}Au_{0.7}/CeO_2(111)$ catalyst. Experiments were performed at 500 K with 1 Torr of methane, while the water pressure was varied from 1 Torr (1:1) to 2 Torr (1:2) and 3 Torr (1:3). Higher water content increases the likelihood of full methane combustion, shifting selectivity from methanol production (reaction 1) to CO/CO_2 formation (reaction 3).

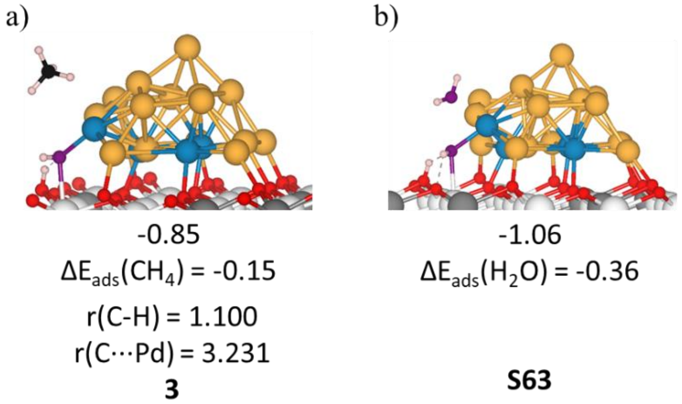

Figure S28. DFT-optimized structures for (a) CH_4 and (b) H_2O adsorbed on the *OH + *H structure **S62**. Energies (in eV) are referenced to the Pd atom model with CH_4 (gas) + H_2O (gas) and $2H_2O$ (gas), respectively. The corresponding adsorption energies of CH_4 and H_2O, respectively, on structure **S62** are also shown. Color scheme: Ce^{4+} (white), Ce^{3+} (gray), O (red), Pd (blue), Au (gold), O from H_2O (purple), H (small white atoms), C (black).

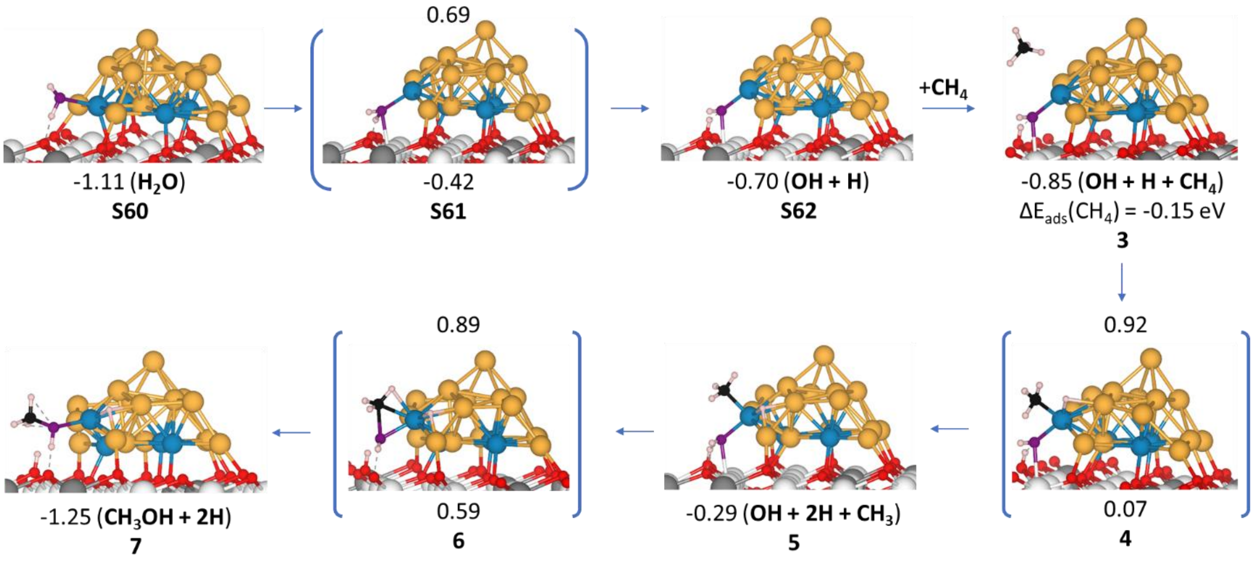

Figure S29. DFT-optimized structures for the H_2O + CH_4 reaction to methanol on the Pd atom model. Energies (in eV) are referenced to the Pd atom model + CH_4 (gas) + H_2O (gas). Activation energies are indicated above the transition state structures. Color scheme: Ce^{4+} (white), Ce^{3+} (gray), O (red), Pd (blue), Au (gold), O from H_2O (purple), H (small white atoms), C (black).

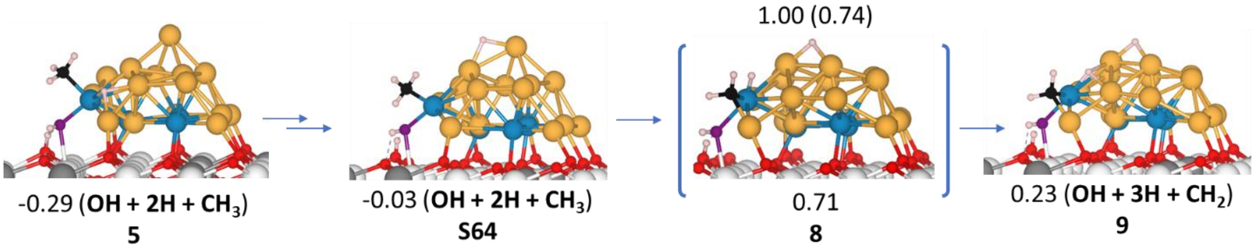

Figure S30. DFT-optimized structures of the competitive CH_3 dissociation reaction on the Pd atom model. Energies (in eV) are referenced to the Pd atom model + CH_4 (gas) + H_2O (gas). Activation energies are indicated above the transition state structures, with values referenced to structure **5** (and referenced to structure **S64** in parentheses). The difference between structures **5** and **S64** lies in the position of the adsorbed H on the cluster. In structure **S64**, the H atom has been moved away from the active Pd site to create space for the reaction.

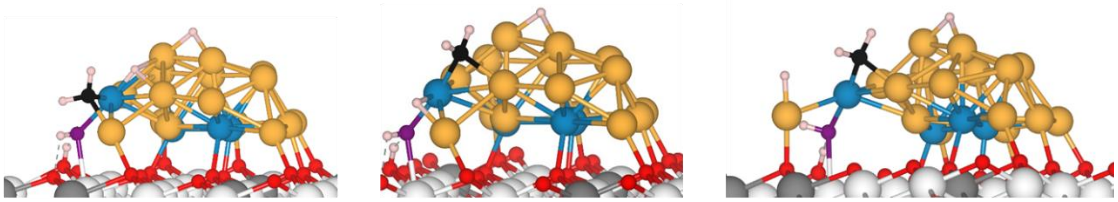

0.23 **9** | 0.34 **S65** | 0.65 **S66**

Figure S31. DFT-optimized structures for *OH + 3*H + $*CH_2$ structures for the Pd atom model. Energies (in eV) are referenced to the Pd atom model + CH_4 (gas) + H_2O (gas). Structure **S66** was initially configured with the H atom on the Au-Au edge but, during optimization, it evolved into an unstable structure, where the Au−Au bond broke and the H atom became monocoordinated. Color scheme: Ce^{4+} (white), Ce^{3+} (gray), O (red), Pd (blue), Au (gold), O from H_2O (purple), H (small white atoms), C (black).
